# A high-throughput method for unbiased quantitation and categorization of nuclear morphology

**DOI:** 10.1093/biolre/ioz013

**Published:** 2019-02-11

**Authors:** Benjamin Matthew Skinner, Claudia Cattoni Rathje, Joanne Bacon, Emma Elizabeth Philippa Johnson, Erica Lee Larson, Emily E K Kopania, Jeffrey Martin Good, Gullalaii Yousafzai, Nabeel Ahmed Affara, Peter James Ivor Ellis

**Affiliations:** 1 Department of Pathology, University of Cambridge, Cambridge, UK; 2 School of Biosciences, University of Kent, Canterbury, UK; 3 Department of Biological Sciences, University of Denver, Denver, CO, USA; 4 Division of Biological Sciences, University of Montana, MT, USA

**Keywords:** spermatogenesis, morphometrics, fertility, image analysis, rodents

## Abstract

The physical arrangement of chromatin in the nucleus is cell type and species-specific, a fact particularly evident in sperm, in which most of the cytoplasm has been lost. Analysis of the characteristic falciform (“hook shaped”) sperm in mice is important in studies of sperm development, hybrid sterility, infertility, and toxicology. However, quantification of sperm shape differences typically relies on subjective manual assessment, rendering comparisons within and between samples difficult.

We have developed an analysis program for morphometric analysis of asymmetric nuclei and characterized the sperm of mice from a range of inbred, outbred, and wild-derived mouse strains. We find that laboratory strains have elevated sperm shape variability both within and between samples in comparison to wild-derived inbred strains, and that sperm shape in F1 offspring from a cross between CBA and C57Bl6J strains is subtly affected by the direction of the cross. We further show that hierarchical clustering can discriminate distinct sperm shapes with greater efficiency and reproducibility than even experienced manual assessors, and is useful both to distinguish between samples and also to identify different morphological classes within a single sample.

Our approach allows for the analysis of nuclear shape with unprecedented precision and scale and will be widely applicable to different species and different areas of biology.

## Teaser

Subtle morphological differences in sperm nuclei can be detected with a new analysis technique; in mice, C57Bl6 and CBA crosses are intermediate to their parental shapes, and the direction of the cross matters.

## Introduction

Cell nuclei are complex, dynamic structures that can adopt a wide range of shapes beyond simply spherical [1]. One of the most profound changes to nuclear shape occurs in spermatogenesis, during which the nucleus successively reshapes and condenses [2, 3]. Most rodents, including mice, have elaborate falciform “hook-shaped” sperm, with varying degrees of hook length and body shape between species [4]. The mouse sperm head shape is established via the interaction of several distinct developmental “modules”, each of which relates to particular cytoskeletal components [5]. When these processes go awry, distinct morphological abnormalities can result [6], linking phenotype with the underlying genetic alterations.

Mouse sperm shape analysis has proven useful in three interrelated areas: evolutionary biology (including speciation), infertility, and toxicology. In evolutionary biology, the questions of how evolutionary forces such as sperm competition and cryptic female choice affect sperm form and function are active fields of research [7, 8], while the high degree of between-species morphological variability means that morphometric analysis can at times aid in species identification [9]. Relatedly, altered regulation of reproductive processes in inter-species hybrids is common, with hybrid males frequently showing highly pleomorphic sperm. The degree to which this morphological instability contributes to speciation-associated process such as hybrid male sterility is also an open question [10]. In particular, in house mouse hybrid sterility, a range of mapped quantitative trait loci (QTL) have been identified on both gonosomes and autosomes that affect both sperm morphology and hybrid sterility [11–14]. In both clinical semen analysis and in mouse knockout models, altered sperm morphology is commonly associated with infertility. However, the role played by specific types and extents of shape defect remains to be elucidated, as does the extent to which teratozoospermia can be used as an indicator of other sperm defects such as DNA damage or defective motility [15]. In toxicology, sperm shape is frequently used as an assessment of genotoxicity and/or reproductive toxicity of compounds [16, 17].

While much sperm analysis still relies on time-consuming and subjective manual scoring, various efforts have been directed towards the development of automated morphometric analyses in an effort to improve both reproducibility and predictive value. To date, these approaches have fallen into three main groups: the measurement of basic parameters such as lengths, widths, and areas of objects; the use of elliptic Fourier analysis to investigate the two dimensional outlines of sperm; and the use of Procrustes analyses to examine differences in fixed landmarks within sperm heads. Each has advantages and disadvantages.

Basic measures such as area (A), length (L), width (W), and perimeter (P) were the first statistics recorded describing sperm morphology [18–20], and still remain useful when an assessment of semen quality must be made rapidly across many different cells [21]. However, such parameters are dominated by the size of the object rather than the shape, and do not allow consistent assessment of the number of normal sperm across populations [22]. Size-independent descriptors can subsequently be constructed from these basic lengths, e.g. L/W ratio (ellipticity) or W/L ratio (aspect ratio). Comparison of linear dimensions to area (e.g. circularity, also known as rugosity or roughness) allows a basic measure of the complexity of the sperm outline, but the values obtained are generic descriptors that cannot be clearly linked to specific elements of the sperm ultrastructure.

More powerful elliptic Fourier descriptors [23] allow an arbitrary closed two dimensional shape to be decomposed into harmonic amplitudes describing the curvature of the object perimeter, allowing subtle variations in shape to be discovered [24]. This has proved powerful for demonstrating differences between species, between laboratory strains, and different experimental treatments [25–27], but has the drawback that both the shape parameters and the underlying mathematics are difficult for biologists to understand and relate back to the biological structure that is affected [28]. Moreover, since Fourier analyses rely on smooth harmonic deformations of an underlying elliptical outline, sharp points—such as found at the tip of a mouse sperm—tend to be poorly fitted [29].

The third major method, Procrustes-based geometric morphometric analysis, uses landmarks and semilandmarks within the object to align individual samples to consistent size, position, and orientation [4, 30]. Principal component analysis can then be used to identify the major varying landmarks distinguishing samples [5]. This approach has the advantage of relating the measured variation to physical structures within the object; however, since objects are aligned by a least-squares method rotating about the centroid, objects are susceptible to smearing of landmarks in highly variable regions, and usually require time-consuming manual placement of landmarks.

To address the unmet need for rapid, unbiased measurement, analysis, and categorization of nuclear morphologies, we have developed a new image analysis tool that automates object finding, alignment, landmark discovery, and sample comparison. This generates quantitative information on the underlying regions of the nucleus that differ within and between samples, independent of nuclear size. Here, we demonstrate the use of this software for each of these approaches by comparing a range of different inbred, outbred, and wild-derived mouse strains, quantifying the morphological variation in highly pleomorphic BALB/c sperm samples, and tracing the genetic influences on sperm morphology in a reciprocal F1 cross between CB57Bl6 and CBA strains.

## Materials and methods

### Mouse strains

All animal procedures were in accordance with the United Kingdom Animal Scientific Procedures Act 1986 and the University of Montana Institute for Animal Care and Use Committee (protocol 002-13) and were subject to local ethical review. Animals were sourced either from an approved supplier (Charles River Laboratories, Manston, UK), bred at Cambridge University Central Biomedical Services (Home Office licenses 80/2451 and 70/8925 held by PE), or bred at the University of Montana (Table 1). Breeding colonies at the University of Montana were established from mice purchased from Jackson Laboratories (Bar Harbor, ME) or were acquired from Francois Bonhomme (University of Montpellier). Animals were housed singly or in small groups, sacrificed via CO_2_ followed by cervical dislocation (UM) or only cervical dislocation and tissues collected post mortem for analysis.

**Table 1.** Mouse strains analyzed in this study. (a) CRL; Charles River Laboratories, Manston, UK; (b) F1 cross animals bred at CRL: B6CBA are routinely available, CBAB6 was set up as a custom request. (c) These are an MF1 outbred strain carrying a Y chromosome derived from RIII strain. Males were obtained from Dr Paul Burgoyne (NIMR) in 2013 and the strain subsequently maintained in Cambridge animal facilities. MF1 females to maintain this strain were sourced from CRL.

Strain Name	Sample ID	Note	Samples imaged	Source (a)
C57Bl6/J	C57Bl6	Inbred	2 individual animals (C57 3, 4)	CRL
CBA/Ca	CBA	Inbred	3 individual animals (CBA1, 2, 3)	CRL
B6CBAF1/Crl (b)	B6CBA	F1 offspring of C57Bl6 (♀) and CBA (♂)	3 individual animals (B6CBA 1, 2, 4)	CRL
CBAB6F1/Crl (b)	CBAB6	F1 offspring of CBA (♀) and C57Bl6 (♂)	4 individual animals (CBAB6 1, 2, 3, 4)	CRL
CRL:CD-1	CD1	Outbred	1 pool of 15 males	CRL
DBA/1J	DBA	Inbred	2 individual animals (DBA 1, 2)	CRL
BALB/cAnNCrl	BALB/c	Inbred	2 individual animals (Balbc 1, 2)	CRL
FVB/N	FVB	Inbred	2 individual animals (FVB 1, 2)	CRL
MF1YRIII (c)	MF1YRIII	Outbred	2 pools (MF1YRIII 1, 2) of 8 males each	Bred at Uni. Cambridge
LEWES/EiJ	LEWES	*M. m. domesticus* Wild-derived inbred	2 pools (LEW 1, 2) of 2 males each	Bred at Uni. Montana
PWK/PhJ	PWK	*M. m. musculus* Wild-derived inbred	2 pools (PWK 2, 3) of 2 males each	Bred at Uni. Montana
STF	STF	*M. spretus* Wild-derived inbred	2 pools (STF 1, 2) of 2 males each	Bred at Uni. Montana

### Sperm collection and fixation

The vasa deferentia and caudae epididymides were dissected from each animal, and the contents squeezed out into 1 mL PBS (scaled accordingly if multiple animals were pooled). Sperm were transferred to a microfuge tube, and tissue clumps were allowed to settle. Sperm were transferred to a new tube and pelleted at 500 g for 5min. The supernatant was removed, and sperm fixed dropwise with either 3:1 methanol-acetic acid or 2% paraformaldehyde (PFA) in PBS. Sperm were pelleted at 500 g for 5min, washed in fixative twice more, then stored at –20°C (methanol-acetic acid) or 4°C (PFA).

### Imaging

Samples were diluted in fixative as required to obtain an evenly spread preparation, and 8 μl of sample dropped onto a slide and allowed to air dry. Slides were stained with 16 μl VectorShield with DAPI (Vector Labs) under a 22 × 50 mm cover slip and imaged using an Olympus UPFLN100XOI2 100× oil immersion plan semiapochromat objective (NA 1.30) on an Olympus BX-61 epifluorescence microscope equipped with a Hamamatsu Orca-ER C4742-80 cooled CCD camera and appropriate filters. Images were captured using Smart-Capture 3 (Digital Scientific, UK). To validate the reproducibility of the software, sample images were also gathered on three other microscopes: (1) an Olympus BX61 with a Hamamatsu C10600 orca r² camera, (2) an Olympus BX61 with a Hamamatsu Orca-03G camera (both (1) and (2) using an Olympus UPFLN100 × 100× oil immersion plan semiapochromat objective [NA 1.30]), and (3) a Nikon Microphot-SA epifluorescence microscope using a Nikon 100× oil immersion plan apochromat objective (NA 1.40) with a Photometrics Metachrome II CH250 cooled CCD camera.

### Nucleus detection and morphological analysis

Image analysis was performed using a custom program designed as a plugin for the freely available image analysis program ImageJ [31]. The software is available at http://bitbucket.org/bmskinner/nuclear_morphology/wiki/Home/ together with full installation instructions, an online wiki user manual, and example testing images. Analyses were conducted using software version 1.14.1. The software allows a user to select a folder of TIFF images previously captured using a fluorescence microscope, and interactively define the nucleus detection parameters. The program then automatically detects and analyses the nuclei in the images.

**Figure 1.**
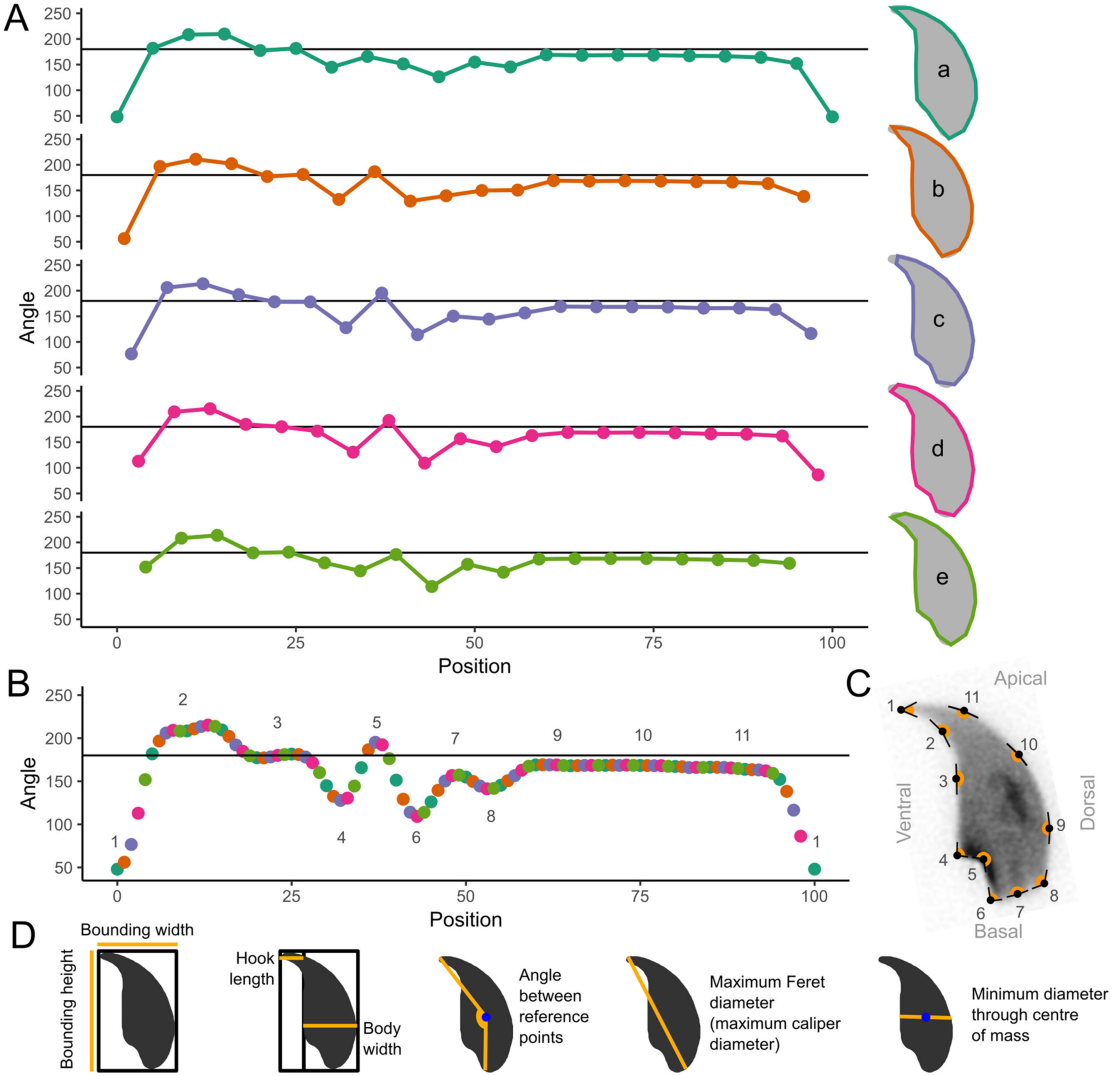
Landmarks are detected by measuring the internal angles around the periphery of the nuclei. (A) The ZR method is based on approximating the true curved shape as a lower resolution polygon with fixed side lengths. The same underlying curve can be encoded multiple ways (a–e) depending on where the vertices of the polygon fall in relation to the underlying shape. For example, the tip is detected well in (a), but not the tail socket; the reverse is true in (d). No individual encoding captures all nuclear features. (B) We measure the angle at every individual pixel around the original shape. This method combines the data from every possible polygonal approximation into a single unified trace, from which landmark features can be detected. (C) Features are marked on a nucleus: 1—tip; 2—under-hook concavity; 3—vertical; 4—ventral angle; 5—tail socket; 6—caudal bulge; 7—caudal base; 8—dorsal angle; 9–11—acrosomal curve. (D) Definitions of key measured parameters used in the software from Table 2. The nucleus center-of-mass is a blue dot. Automatic vertical orientation is used to determine bounding dimensions.

Once nuclei are acquired from a set of images, they are consistently oriented and aligned. Landmarks are automatically identified using a modification of the Zahn-Roskies (ZR) transform [32] to generate a linear trace we refer to as the angle profile (Figure 1). The conventional ZR transform approximates a given shape as a polygon based on a fixed number of semilandmarks spaced evenly around the perimeter of the shape, and then measures the angle at each vertex of the resulting polygon [4]. In our analyses, we measure the interior angle across a window of 5% of the total object perimeter—equivalent to a ZR transform with 20 semilandmarks per object. This window size was chosen to be maximally informative, following testing of a range of values (Supplementary Figure S4). However, in contrast to the ZR transform that uses a single set of semilandmarks per object and only measures the angle at each semilandmark, we instead measure the interior angle at every point around the shape's perimeter. The final result is thus equivalent to combining multiple overlapping ZR transforms, each offset by a single point so as not to duplicate or lose information. We find this to give a higher resolution encoding of the shape that loses less information in finely detailed areas such as the hook tip and tail attachment site.

The angle profiles from each nucleus are interpolated to a consistent length and aligned against other. A median profile is constructed by taking the median of the angles at each point along the interpolated profile length. The median profile is segmented at local mimima and local maxima below or above 180 degrees respectively, automatically defining landmarks at convex or concave corners in the shape. The landmark locations in each nucleus are then identified via the best fit of the nucleus profile to the median at each landmark. The flat region below the hook is used to allow consistent vertical orientation of the nuclei. A diagram of the full analysis pipeline is provided as Supplementary Figure S1.

### Statistical analysis and clustering

Following segmentation, standard nuclear parameters are automatically measured: area, perimeter, and ellipticity, the width of the nuclear body versus the length of the hook as described in other papers [20], and the lengths of each perimeter segment (Table 2; Figure 1D). In order to quantify the variability of the nuclear shapes, we developed a new per-nucleus measure defined as the root-mean-square difference between the per-nucleus angle profile and the median angle profile for the dataset, after interpolation to a fixed length. Summary statistics are automatically calculated.

**Table 2.** Parameters measured in the software.

Parameter	Description
Area	A; the two-dimensional area of the nucleus
Perimeter	P; the length of the nuclear perimeter
Max feret diameter	the maximum caliper diameter across the nucleus
Min diameter	the shortest caliper diameter through the center of mass of the nucleus
Variability	√(((∑(d²))/L); the square root of the sum-of-squares difference (d) at each index between the nuclear profile and the dataset median profile, after normalization to a fixed length (L)
Ellipticity	H/W; the height (H) divided by width (W) of the nuclear bounding box when the nucleus is vertically oriented
Circularity	4πA/P²; the closeness of the nucleus to a circle, between 0 and 1, where 1 is a perfect circle.
Bounding width	W; the width of the bounding rectangle of the vertically oriented nucleus
Bounding height	H; the height of the bounding rectangle of the vertically oriented nucleus
Angle between reference points	the angle between the tip, the centre of mass, and the caudal reference point (defined as the point of greatest curvature at the rear of the sperm head)
Length of hook [rodent sperm only]	the distance from the vertical alignment region to the x-edge of the bounding rectangle on the hook side (Figure 1D)
Width of body [rodent sperm only]	the distance from the vertical region to the x-edge of the bounding rectangle on the body side (Figure 1D)
Segment lengths	the length of each segment along the perimeter of the nucleus

Data were exported for further processing in R. Differences between mouse strains were tested using a pairwise Wilcoxon rank sum test, with Bonferroni multiple testing correction. The coefficient of variability (standard deviation/mean) was also calculated for each of the other measured parameters.

The “average shape” of the nuclei was calculated by averaging the x and y coordinates at consistent semilandmarks spaced every 1% of the perimeter across all nuclei, vertically aligned and with their centers of mass at (0,0). This yielded a “consensus nucleus” visualizing the overall shape of the population. Clustering was implemented via the WEKA data mining software library [33].

## Results

### Morphology analysis is robust to image capture conditions

Before investigating the biological differences between samples, we needed to be confident that our analyses were reproducible and not biased by small differences in data gathering—for example, the camera and microscope used to capture images, and the exposure time during image capture. The choice of fixative (3:1 methanol:acetic acid [MeAc] vs 2% PFA) did not affect overall shape (Supplementary Figure S7), but had a minor and inconsistent effect on sperm head area (Supplementary Figure S8). We standardized on paraformaldehyde fixation for the remainder of our analyses. The objective lens and camera did not affect our results (Supplementary Figure S9), and automatic exposure time produced images equal to an optimized fixed exposure time (Supplementary Figure S10). We standardized on data from a single microscope using automatic exposure times for the subsequent image capturing.

### Detection and quantification of sperm shape in C57Bl6 and CBA mice

**Figure 2.**
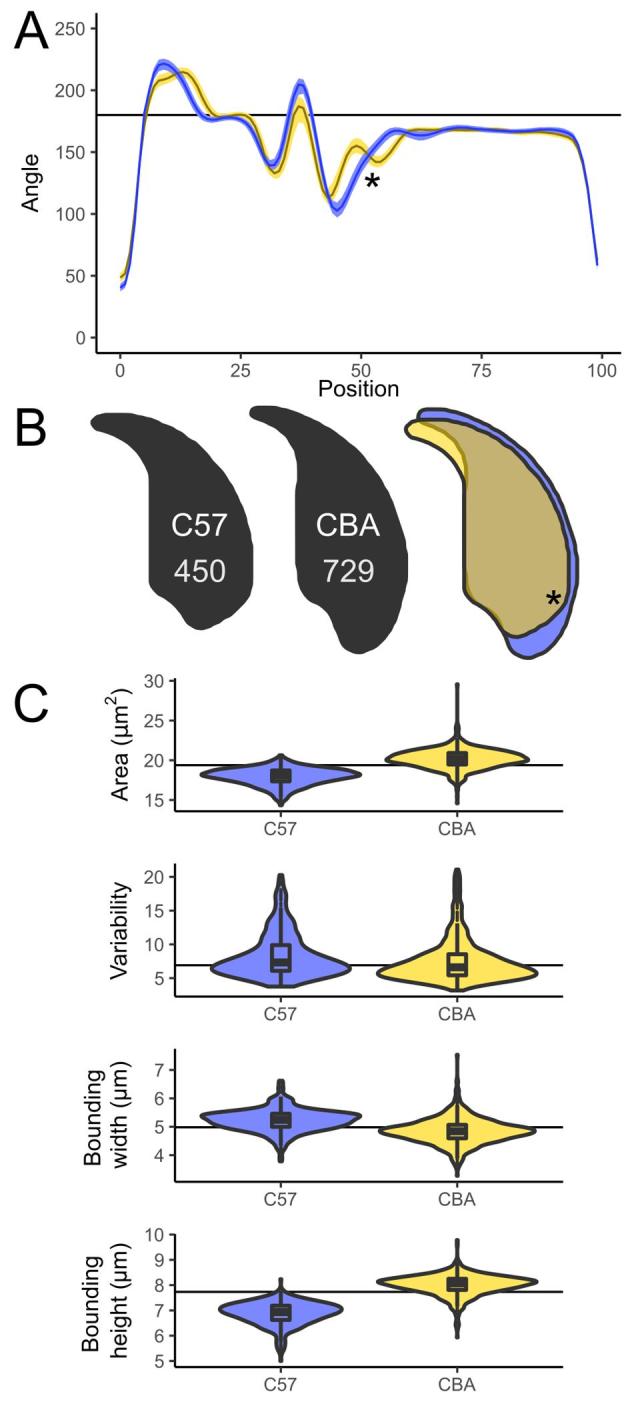
(A) Comparison of angle profiles between C57Bl6 (yellow) and CBA (blue), showing the median and interquartile range of the nuclear angle profiles. (B) Consensus nuclei from each population, and the overlap showing the regions differing. (C) Size and shape measurements between the strains; sperm numbers are shown on the consensus nuclei. The prominent dorsal angle in C57Bl6 nuclei is marked with an asterisk.

CBA and C57Bl6 sperm are distinguishable to the trained eye, and make a useful demonstration of the software, as the angle profiles generated are distinct for each genotype (Figure 2A). CBA sperm have a larger cross-sectional area, are longer, and also have slightly shorter hooks than C57Bl6 sperm (Figure 2B and C). These differences are reflected in the profiles; the long narrow tail in the CBAs appears as a smooth curve at x = 50 in the profile, while the shorter, wider C57Bl6s show a distinct dip corresponding to the sharper curve of the dorsal angle before the acrosome. The shorter hook of the CBAs is also seen as a narrow peak at x = 10; a detailed comparison of segmentation patterns is given in Supplementary Figure S11.

CBA and C57Bl6 have previously been characterized by Wyrobek et al. [20], who measured 160 nuclei of each genotype by manual tracing of projected microscope images of eosin-stained sperm heads. We found our measured values to be similar (Supplementary Table S6) but slightly smaller—as expected given that their measurements are for the entire sperm head rather than just the nucleus. We measured the CBAs to be 12% longer than the C57Bl6s, again close to the previously published 13.5%.

### The degree of intra-sample morphological variability is affected both by inbreeding and inter-species hybridization

**Figure 3.**
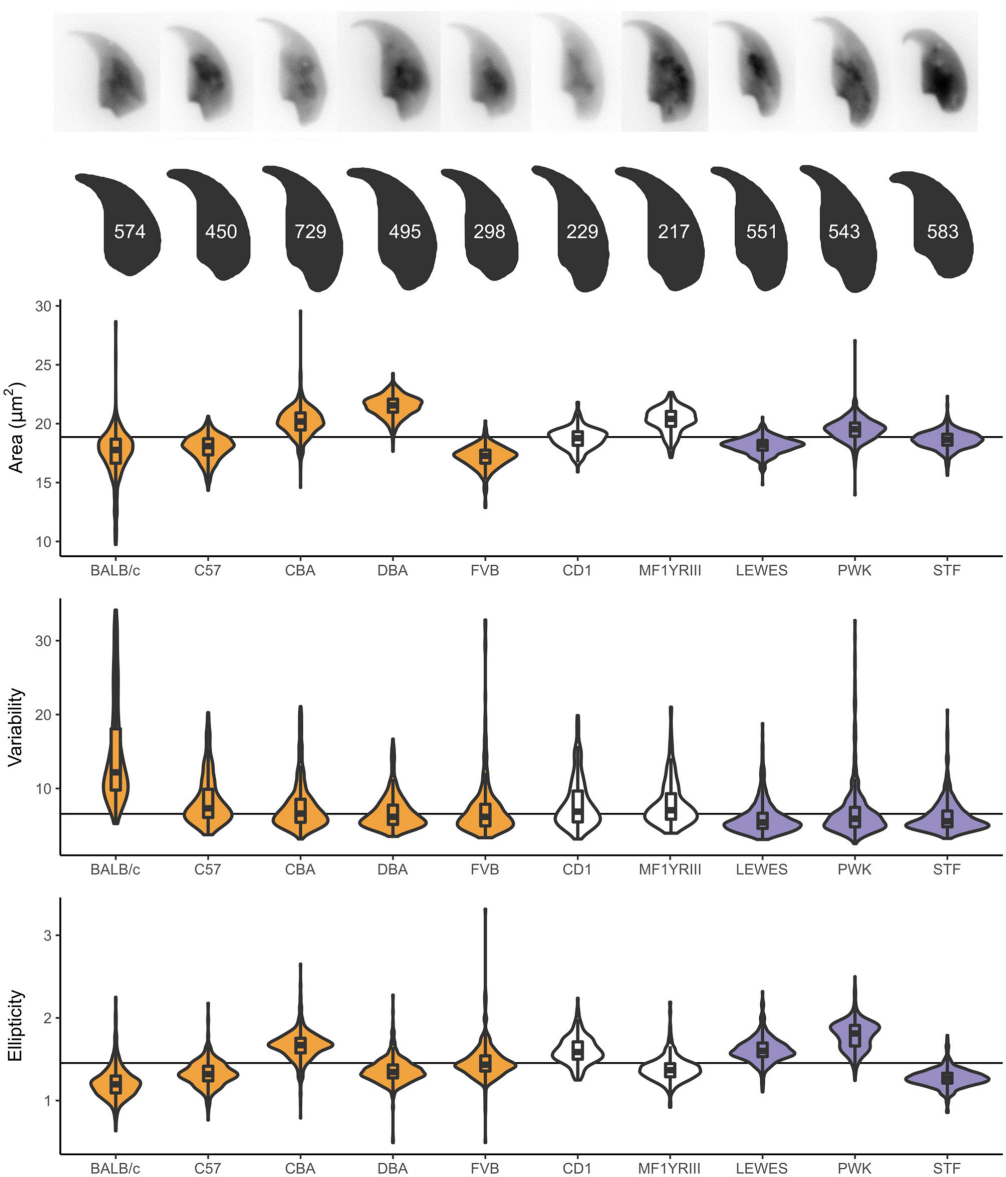
Parameters for additional strains examined, with representative nuclei and population consensus nucleus showing the number of analyzed sperm. Samples are colored according to their type: from left to right: inbred (yellow), outbred (white), and inbred wild-derived (blue).

Having demonstrated the software can distinguish differences between two genotypes, we carried out a preliminary investigation of the extent to which sperm shape variability in classical laboratory strains is affected by two factors: inbreeding and the complex inter-subspecific mosaic origin of these strains. To do this, we compared a panel of inbred laboratory strains to (a) outbred laboratory strains, and (b) wild-derived inbred strains (Table 1). Biological replicate samples from the inbred strains represent either single animals (laboratory inbred strains) or a pool of two animals (wild-derived inbred strains). For the outbred strains, several individuals were pooled to sample the diversity across the population. A comparison of the average nuclear shape for each strain is shown in Figure 3. In addition to each strain having a characteristic sperm morphology, different strains showed different levels of intra-sample variability. Importantly, breakdown by biological replicates shows that these data reflect true strain differences rather than sample-specific factors such as technical differences between imaging sessions or choice of fixative (Supplementary Figure S12; Supplementary Table S2).

Some of the automatically identified landmarks were consistently found across all strains, such as the tip of the apical hook and the point of maximum curvature at the base of the sperm head, while others (such as the dorsal angle and the indentation at the tail attachment site) were variable between strains. Of all the strains studied, only five showed a clear dorsal angle, with the others having a smoother profile posterior to the acrosome. The distance from the rear reference point to the dorsal angle was characteristic for each of these five strains, as was the variability in this measurement, with BALB/c mice showing highest variability. Supplementary Figure S13 demonstrates the ubiquitous and variable landmarks discovered by the segmentation analysis and shows the detailed segmentation pattern for each strain, while Supplementary Table S3 gives the numerical segment length data for each strain.

Overall, sperm shape variability within each strain was assessed using a new measure based on the similarity of each cell's angle profile to the median for that strain. This correlated well with other population measures of variability such as the coefficients of variation for area, bounding height and perimeter (Supplementary Table S7). The BALB/c mice have the most variable shape profiles of all the strains we analyzed, as well as the highest coefficient of variability in area, height and width (Figure 3). The other inbred laboratory strains all showed low intra-strain variability despite the fact that there were marked differences in sperm size and shape between strains. Of the inbred laboratory strains tested, CBA and DBA had the lowest intra-sample variability. The two outbred strains, CD1 and MF1Y^RIII^ both showed slightly higher intra-sample variability. This may reflect the fact that these samples were pooled samples derived from multiple genetically unique individuals. Of the wild-derived strains, all three lineages analyzed (*M. m. domesticus, M. m. musculus*, and *M. spretus*) had lower variability than any of the standard laboratory strains, despite that fact that these wild-derived strains are inbred.

### C57Bl6/CBA F1 strain cross males demonstrate the effects of each parental genotype on sperm shape and stabilization of sperm morphology in F1 males

**Figure 4.**
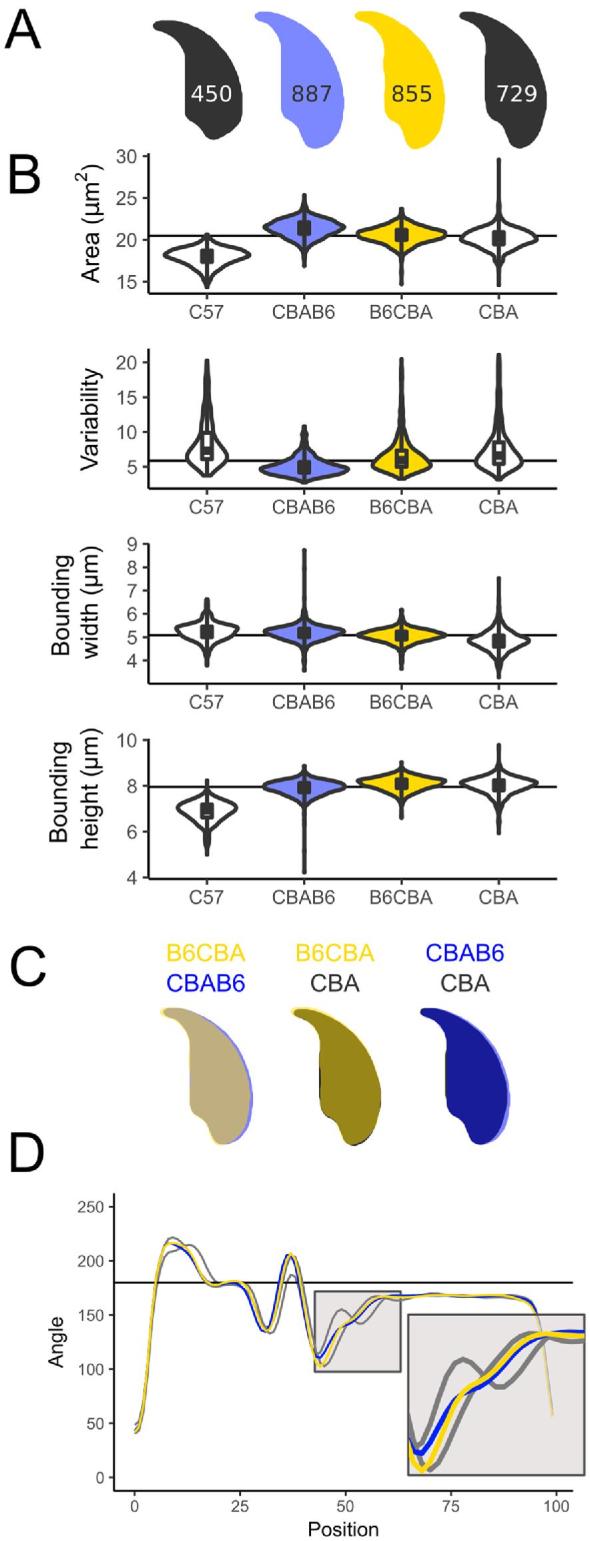
Subtle differences can be seen between a CBAB6 (CBA mother; blue) and a B6CBA (C57Bl6 mother; yellow). Both are intermediate to the parental shapes (grey), but CBAB6 sperm are wider, and their shape is closer to that of the C57Bl6. (A) Consensus nuclei with the number of analyzed sperm; (B) size measurements; (C) overlay of consensus nuclei; (D) comparison of angle profiles; the tail attachment region is expanded in the inset.

We investigated the impact of strain background and genetic interactions using one specific reciprocal cross, between C57Bl6 and CBAs. The use of F1 animals means the resulting animals are no longer inbred, but still yields a uniform population of genetically identical males from each cross. B6CBA mice are the F1 offspring of a female B6 with a male CBA and CBAB6 mice are the reciprocal cross. Sperm morphology for both directions of the cross matches the CBA parental strain closely, indicating a dominant effect of the CBA genotype (Figure 4A), and both types of F1 sperm are much more similar to the CBA parent in cross sectional area (Figure 4B). Consistent with previous work [34], F1 males showed less variability in their sperm shape compared to either parent strain, suggesting that inbreeding acts to destabilize sperm morphology, and this is relieved via heterosis in the F1s.

The reciprocal cross data allows us to look for parent-of-origin effects on sperm shape. We found two differences, in sperm cross-sectional area and in bounding width. CBAB6s have a slightly larger sperm area than the B6CBAs (19.3 vs 18.6 μm^2^, *P* < 0.001) and the region around the posterior of the nucleus is widened in the CBAB6s, intermediate to CBA and C57Bl6 (Figure 4B and C). The differences around the posterior are largely driven by changes in the dorsal angle, which is present in C57, absent in CBA, and virtually absent in both reciprocal F1 cross males (Figure 4D). For bounding width, we find that this parameter is influenced by the male parent: CBAB6 and B6CBA are significantly different to each other (*P* = 0.0016), as are C57Bl6 and CBA (*P* = 1.27E-12), but there is no significant difference between C57Bl6 and CBAB6 (*P* = 0.18) or between CBA and B6CBA (*P* = 0.095). This suggests that this aspect of sperm shape may be influenced either by sex chromosome or mitochondrial background or by autosomal imprinted loci.

### Hierarchical clustering can separate samples based on shape differences

Finally, we investigated the use of unsupervised cluster analysis of sperm shape parameters to detect different morphological sub-populations within a single sample. Using a hierarchical clusterer, we separated sperm based on their shape profiles. Initial testing using data from C57Bl6 and CBA males showed that the clusterer performed at least as well (96% accuracy) as experienced assessors (97% accuracy), and substantially better than novice assessors (75% accuracy) at distinguishing between these two strains (Supplementary Table S4; Supplementary Figure S14).

**Figure 5.**
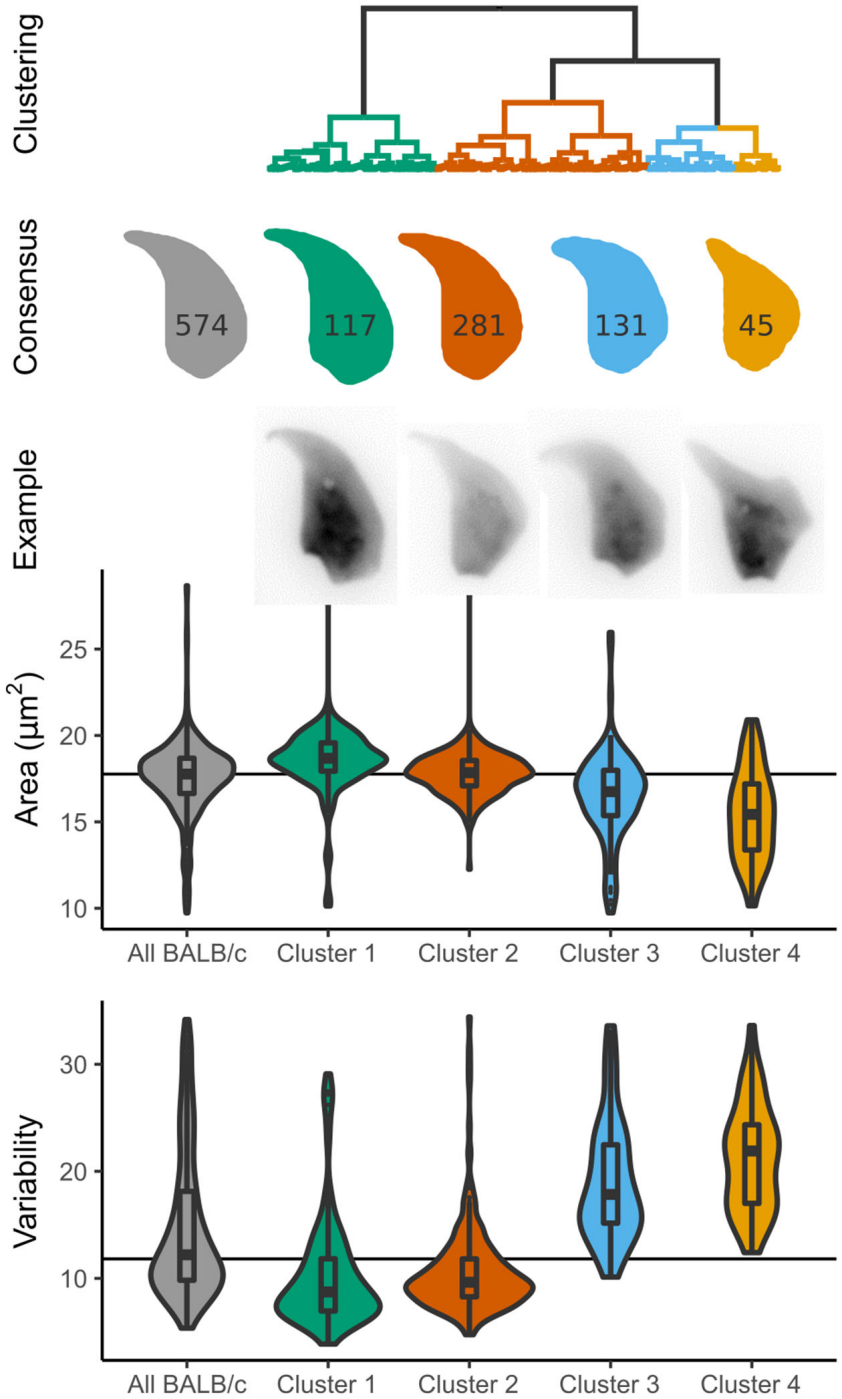
Clustering of BALB/c sperm reveals sub-populations of nuclei. Compared to the overall population of BALB/c sperm (grey), nuclei with distinct shapes are revealed, from mostly normal (green) to highly abnormal (yellow). The number of sperm in each cluster is given on the consensus shape. Sperm from each of the two animals are equally represented in each cluster (Supplementary Table S7).

Next, we looked at using the clustering for novel shape discovery in BALB/c, the strain with highest within-sample variability. Clustering revealed four major groups of sperm shape, from mostly normal sperm to severely shrunken and misshapen sperm (Figure 5). Each of the two BALB/c samples was equally represented in the clusters (Supplementary Table S7), i.e. the clustering procedure can categorize intra-strain morphological variability independent of any individual biological or technical variation between the samples. The final class is still highly variable compared to the other classes; sub-clustering these nuclei further reveals a separation into two groups of highly abnormal sperm (Supplementary Figure S15) as previously described [32]. While the most normal sperm had near-normal placement of the dorsal angle and a normal tail attachment site, the most heavily distorted sperm showed frequent presence of additional sharp angles in the sperm outline, effacement of the tail attachment site due to compression of the rear of the sperm head, and an ever more prominent and misplaced dorsal angle that may reflect altered microtubule dynamics during nuclear shaping.

## Discussion

We present a novel tool for nuclear morphometry, which quantitatively measures a range of nuclear and sub-nuclear size and shape parameters. While we have chosen mouse sperm to demonstrate the software, the analysis steps will work on many symmetric or asymmetric shapes of nuclei including, but not limited to sperm from other species [35].

At the object detection stage, we use an edge detection algorithm that is markedly more effective than fixed-threshold detection. At the shape decomposition step, we introduce a modification of the ZR transform [32] that sensitively detects the various angular landmarks around the nuclear periphery without the need for manual intervention. Together, these key innovations permit automation of the steps involved in object detection, shape decomposition and comparison, massively increasing the number of nuclei that can be quantified and compared to each other. This allows the use of sample numbers that accurately capture not only fixed size and shape differences between samples, but also the detection and classification of intra-sample variability; with a total of 8749 nuclei being measured during this preliminary study.

### Comparison of sperm shape and variability within and between strains

Our observations support previous studies of mouse sperm morphological variation [4, 20], and add further information on the precise regions of the sperm head that that differ between strains. We also demonstrate the variability of sperm morphology within each given strain. In particular, we examined the presence and placement of the dorsal angle of the sperm. This feature is created by pressure from the manchette: a cone-shaped array of microtubules that forms behind the nucleus and slides backwards during spermiogenesis, shaping the rear of the sperm head in the process. Defects in katanin p80, a microtubule severing protein, lead to failure of this process and abnormal compression of the base of the sperm head [6]. The narrowing of the tail attachment site seen in FVB and BALB/c males, together with the prominent dorsal angle seen in both strains (especially the latter) may indicate that manchette migration is altered in these males.

The greatest sperm shape variability was observed in the BALB/c animals, a strain with poor sperm morphology and high levels of sperm aneuploidy. Kishikawa et al. [36] observed different classes of sperm head shape, which we were able to recapitulate. In their analysis, the authors found chromosomal abnormalities in 35% of sperm that were scored as highly abnormal according to their criteria, but also in 15% of sperm that were scored as morphologically normal. Given that our new analysis detects classes with more subtle shape differences, we hypothesize that these new classes may also be enriched for chromosomal defects. Further differences await characterization: different classes of sperm morphology have been described depending on the particular substrain and age of the animal [37].

### Investigating the origin of elevated within-sample variability in laboratory strains

Consistent with [34], we found that an F1 cross between C57Bl6 and CBA laboratory strains lowered sperm shape variability (see below), suggesting that the parental inbred strains have fixed combinations of alleles that lead to less stable sperm morphology. However, the least variable strains we examined were the wild-derived inbred strains PWK, LEW, and STF, representing *M. m. musculus, M. m. domesticus* and *M. spretus* respectively. Since these three strains are also inbred, this suggests that the variety of sperm shapes in laboratory strains, and the elevated level of intra-individual variability in all the laboratory strains is not solely a consequence of inbreeding. Instead, this is potentially linked to the status of the laboratory mouse as a hybrid between several mouse subspecies—a factor that may have disrupted regulatory interactions throughout the genome, particularly interactions involving the sex chromosomes [38–40]. Against this, PWK, despite being predominantly of *musculus* origin, nevertheless has substantial introgression of *domesticus* DNA, of the order of ∼6%–7% of the genome [40, 41]. The degree of disruption may therefore depend on both the direction of introgression and the specific regions involved, and the various different classical and wild-derived inbred strains may have fixed different combinations of incompatible alleles that collectively destabilize sperm development to varying extents in each strain [41].

An alternative but not mutually exclusive explanation for the difference between classical laboratory inbred strains and wild-derived inbred strains is that the classical strains have been selected over multiple generations for their ability to breed well in captivity—indeed FVB is particularly known for its fecundity [42]. Under laboratory conditions of non-competitive mating, co-housing a single male with one or more females, it is likely that reproductive output is driven largely by maternal factors. In strains experimentally selected for high fecundity, male fertility and sperm morphology/motility parameters are compromised, suggestive of a trade-off between the male and female factors necessary for high fecundity in a laboratory environment [43].

### Future uses for our approach in speciation, fertility, and toxicology studies

Sperm morphology is of interest from an evolutionary perspective; sperm are under intense selection, sperm morphology has been found to be an important criterion influencing male fertility in many species [44]. Altered sperm head morphology has emerged as a common form of hybrid male sterility in mice [11–14, 45]. Some sterility factors broadly impair spermatogenesis, resulting in reduced sperm counts and lower motility in addition to head shape alterations. However, several studies have now shown that hybrid sterility QTL in mice often correspond to specific reproductive phenotypes [14]. The challenges of manually quantifying morphology in large mapping panels has necessitated the use of crude categorical scores [11, 13, 45], hampering quantitative precision and limiting the ability to draw causal links between hybrid incompatibilities and specific aspects of sperm morphological development. Our approach assists firstly by enabling more rigorous quantitation of sperm shape, and secondly by enabling the large sample sizes and systematic approach needed for mapping studies.

Fertility rate and IVF efficiency has been correlated with the genetic background of sperm among inbred mouse strains [46]. Furthermore, many studies have shown that the genetic background of a strain can influence sperm morphology. For example, deletion of the long arm of the Y chromosome results in a more severe phenotype on B10.BR background than on CBA [47]. Mashiko et al. [27] have suggested morphology of sperm is associated with fertilizing efficiency in at least two mouse strains (B6D2F1 and C57Bl6/N). Since particular genetic mutations in mouse sperm shape are associated with characteristic nuclear shape alterations [15], detailed examination of sperm from natural mutant and/or targeted knockout animals may point to pathways of interest for understanding spermiogenesis and male fertility more generally.

In toxicological analysis, rodent sperm are conventionally manually classified into classes of predefined morphological abnormality [16, 48]. The hierarchical clustering implemented within the software is able to separate nuclei based on shape as accurately as an experienced manual sperm scorer, and is faster and more consistent. This may be of use in samples where the nature and degree of abnormalities is hard for humans to reliably quantify—e.g., where the shape defects seen do not match existing scoring charts. The fact that specific genetic lesions cause specific shape changes means that the sperm shape might in principle give information not just about the presence/absence of toxicity but also its mode of action. Our new analysis approach will complement existing studies of sperm function, which, in clinical settings or in automated CASA platforms [49], is still lacking detailed morphological data [21].

## Supplementary data

**Supplementary Methods and Results:** Detailed description of the analysis methods and software validation.

**Supplementary Table S1.** The mean measured parameters by strain, with coefficient of variability, standard error, and standard deviation per parameter

**Supplementary Table S2:** The mean measured parameters by individual sample, with coefficient of variability, standard error, and standard deviation per parameter

**Supplementary Table S3:** The default mean segment lengths by strain, with coefficient of variability, standard error, and standard deviation per segment

ioz013_Supplemental_Files

## Notes

Edited by Dr. Monika A. Ward
