# Supplementary material for: A high-throughput method for unbiased quantitation and categorization of nuclear morphology
Source: Biol Reprod. 2019 Feb 11;100(5):1250–60. doi: 10.1093/biolre/ioz013 (PMC6497523; doi:10.1093/biolre/ioz013)
Supplement: ioz013_Supplemental_Files [file ioz013_supplemental_files.zip › Supplementary table 1.pdf]

Supplementary Table 1: Parameters measured by the software across samples, grouped by strain

				Angle between reference points (degrees)			
Strain	Status	Fixative	Number of nuclei analysed	Coefficient of Variation	Mean	Standard error of the mean	Standard Deviation
BALB/c	Inbred	MeAc	420	8.45	142	0.584	12
BALB/c	Inbred	PFA	574	5.59	141	0.329	7.87
C57	Inbred	MeAc	505	3.07	140	0.192	4.31
C57	Inbred	PFA	450	1.94	139	0.127	2.69
CBA	Inbred	MeAc	242	7.08	148	0.673	10.5
CBA	Inbred	PFA	729	1.64	147	0.0888	2.4
DBA	Inbred	PFA	495	1.98	139	0.124	2.75
FVB	Inbred	MeAc	216	7.91	149	0.802	11.8
FVB	Inbred	PFA	298	2.55	148	0.218	3.76
B6CBA	F1 hybrid	PFA	855	1.58	143	0.0769	2.25
CBAB6	F1 hybrid	PFA	887	1.29	143	0.0619	1.84
CD1	Outbred	MeAc	260	3.08	147	0.281	4.54
CD1	Outbred	PFA	229	2.2	143	0.208	3.15
MF1YRIII	Outbred	MeAc	277	7.09	141	0.599	9.97
MF1YRIII	Outbred	PFA	217	2.19	139	0.207	3.05
LEWES	Wild-derived	PFA	551	1.37	147	0.0863	2.03
PWK	Wild-derived	PFA	543	1.7	152	0.111	2.58
STF	Wild-derived	PFA	583	2.24	137	0.127	3.07

				Area (square microns)			
Strain	Status	Fixative	Number of nuclei analysed	Coefficient of Variation	Mean	Standard error of the mean	Standard Deviation
BALB/c	Inbred	MeAc	420	21.6	18.1	0.191	3.91
BALB/c	Inbred	PFA	574	12	17.5	0.0877	2.1
C57	Inbred	MeAc	505	7.19	19.2	0.0615	1.38
C57	Inbred	PFA	450	5.86	17.9	0.0496	1.05
CBA	Inbred	MeAc	242	12.6	21	0.171	2.65
CBA	Inbred	PFA	729	6.09	20.2	0.0455	1.23
DBA	Inbred	PFA	495	4.21	21.5	0.0406	0.903
FVB	Inbred	MeAc	216	20.9	16.8	0.239	3.51
FVB	Inbred	PFA	298	5.53	17.2	0.055	0.949
B6CBA	F1 hybrid	PFA	855	4.77	20.6	0.0335	0.98
CBAB6	F1 hybrid	PFA	887	5.02	21.4	0.036	1.07
CD1	Outbred	MeAc	260	15.3	18.5	0.175	2.82
CD1	Outbred	PFA	229	4.81	18.8	0.0596	0.902
MF1YRIII	Outbred	MeAc	277	23.6	21.8	0.309	5.14
MF1YRIII	Outbred	PFA	217	5.17	20.3	0.0713	1.05
LEWES	Wild-derived	PFA	551	3.91	18.1	0.0302	0.709
PWK	Wild-derived	PFA	543	4.9	19.5	0.041	0.956
STF	Wild-derived	PFA	583	4.08	18.6	0.0315	0.761

				Aspect ratio			
Strain	Status	Fixative	Number of nuclei analysed	Coefficient of Variation	Mean	Standard error of the mean	Standard Deviation
BALB/c	Inbred	MeAc	420	19.4	0.861	0.00815	0.167
BALB/c	Inbred	PFA	574	15.5	0.844	0.00546	0.131
C57	Inbred	MeAc	505	11.9	0.795	0.00422	0.0948
C57	Inbred	PFA	450	12.1	0.766	0.00438	0.0928
CBA	Inbred	MeAc	242	10.6	0.585	0.00398	0.0618
CBA	Inbred	PFA	729	11.4	0.607	0.00257	0.0693
DBA	Inbred	PFA	495	14.4	0.746	0.00482	0.107

FVB	Inbred	MeAc	216	31.6	0.614	0.0132	0.194
FVB	Inbred	PFA	298	16.3	0.696	0.00656	0.113
B6CBA	F1 hybrid	PFA	855	6.81	0.627	0.00146	0.0427
CBAB6	F1 hybrid	PFA	887	11.9	0.658	0.00262	0.078
CD1	Outbred	MeAc	260	17.8	0.603	0.00666	0.107
CD1	Outbred	PFA	229	10.1	0.629	0.00422	0.0638
MF1YRIII	Outbred	MeAc	277	15	0.74	0.00666	0.111
MF1YRIII	Outbred	PFA	217	10.6	0.731	0.00526	0.0775
LEWES	Wild-derived	PFA	551	9.36	0.625	0.00249	0.0585
PWK	Wild-derived	PFA	543	6.19	0.51	0.00135	0.0315
STF	Wild-derived	PFA	583	8.94	0.792	0.00293	0.0709

				**Bounding height (microns)**			
Strain	Status	Fixative	Number of nuclei analysed	Coefficient of Variation	Mean	Standard error of the mean	Standard Deviation
BALB/c	Inbred	MeAc	420	15.1	6.5	0.0478	0.979
BALB/c	Inbred	PFA	574	9.89	6.4	0.0264	0.634
C57	Inbred	MeAc	505	8.55	6.84	0.026	0.585
C57	Inbred	PFA	450	6.58	6.88	0.0213	0.452
CBA	Inbred	MeAc	242	10.5	8.09	0.0545	0.847
CBA	Inbred	PFA	729	5.07	8	0.015	0.405
DBA	Inbred	PFA	495	5.56	7.72	0.0193	0.429
FVB	Inbred	MeAc	216	15.8	7.4	0.0795	1.17
FVB	Inbred	PFA	298	5.28	7	0.0214	0.37
B6CBA	F1 hybrid	PFA	855	3.6	8.09	0.00996	0.291
CBAB6	F1 hybrid	PFA	887	4.25	7.9	0.0113	0.336
CD1	Outbred	MeAc	260	10.8	8.04	0.0537	0.866
CD1	Outbred	PFA	229	4.53	7.95	0.0238	0.361
MF1YRIII	Outbred	MeAc	277	13.5	7.95	0.0643	1.07
MF1YRIII	Outbred	PFA	217	4.17	7.66	0.0217	0.32
LEWES	Wild-derived	PFA	551	4.06	7.82	0.0135	0.317
PWK	Wild-derived	PFA	543	3.87	8.19	0.0136	0.317
STF	Wild-derived	PFA	583	3.8	6.64	0.0105	0.253

				**Bounding width (microns)**			
Strain	Status	Fixative	Number of nuclei analysed	Coefficient of Variation	Mean	Standard error of the mean	Standard Deviation
BALB/c	Inbred	MeAc	420	16.8	5.51	0.0452	0.926
BALB/c	Inbred	PFA	574	12.3	5.35	0.0274	0.657
C57	Inbred	MeAc	505	7.51	5.4	0.018	0.405
C57	Inbred	PFA	450	7.55	5.23	0.0186	0.395
CBA	Inbred	MeAc	242	9.23	4.7	0.0279	0.434
CBA	Inbred	PFA	729	9.24	4.85	0.0166	0.448
DBA	Inbred	PFA	495	7.27	5.73	0.0187	0.416
FVB	Inbred	MeAc	216	21.1	4.45	0.0638	0.937
FVB	Inbred	PFA	298	10.2	4.85	0.0286	0.494
B6CBA	F1 hybrid	PFA	855	5.62	5.06	0.00973	0.285
CBAB6	F1 hybrid	PFA	887	6.78	5.18	0.0118	0.351
CD1	Outbred	MeAc	260	17.9	4.82	0.0536	0.865
CD1	Outbred	PFA	229	7.92	4.99	0.0261	0.395
MF1YRIII	Outbred	MeAc	277	18.2	5.86	0.0641	1.07
MF1YRIII	Outbred	PFA	217	7.61	5.58	0.0288	0.425
LEWES	Wild-derived	PFA	551	6.96	4.87	0.0144	0.339
PWK	Wild-derived	PFA	543	8.09	4.61	0.016	0.373
STF	Wild-derived	PFA	583	6.19	5.25	0.0134	0.325

**Circularity**			

Strain	Status	Fixative	Number of nuclei analysed	Coefficient of Variation	Mean	Standard error of the mean	Standard Deviation
BALB/c	Inbred	MeAc	420	13.7	0.549	0.00366	0.0749
BALB/c	Inbred	PFA	574	9.64	0.566	0.00228	0.0546
C57	Inbred	MeAc	505	8.24	0.575	0.00211	0.0474
C57	Inbred	PFA	450	5.78	0.539	0.00147	0.0312
CBA	Inbred	MeAc	242	9.88	0.555	0.00352	0.0548
CBA	Inbred	PFA	729	6.32	0.53	0.00124	0.0335
DBA	Inbred	PFA	495	4.03	0.53	0.00096	0.0214
FVB	Inbred	MeAc	216	14.3	0.502	0.0049	0.072
FVB	Inbred	PFA	298	6.04	0.539	0.00189	0.0326
B6CBA	F1 hybrid	PFA	855	5.09	0.517	0.000899	0.0263
CBAB6	F1 hybrid	PFA	887	3.8	0.545	0.000695	0.0207
CD1	Outbred	MeAc	260	9.35	0.492	0.00286	0.046
CD1	Outbred	PFA	229	6.42	0.487	0.00207	0.0313
MF1YRIII	Outbred	MeAc	277	14.4	0.498	0.00431	0.0717
MF1YRIII	Outbred	PFA	217	4.78	0.507	0.00164	0.0242
LEWES	Wild-derived	PFA	551	4.11	0.495	0.000865	0.0203
PWK	Wild-derived	PFA	543	4.58	0.518	0.00102	0.0237
STF	Wild-derived	PFA	583	3.91	0.577	0.000935	0.0226

				**Variability**			
Strain	Status	Fixative	Number of nuclei analysed	Coefficient of Variation	Mean	Standard error of the mean	Standard Deviation
BALB/c	Inbred	MeAc	420	61.5	16.8	0.505	10.4
BALB/c	Inbred	PFA	574	46.6	14.7	0.287	6.87
C57	Inbred	MeAc	505	72.6	9.21	0.298	6.69
C57	Inbred	PFA	450	39.9	8.35	0.157	3.33
CBA	Inbred	MeAc	242	72.3	9.16	0.426	6.62
CBA	Inbred	PFA	729	42.9	7.56	0.12	3.24
DBA	Inbred	PFA	495	33.8	6.75	0.102	2.28
FVB	Inbred	MeAc	216	81	15.6	0.86	12.6
FVB	Inbred	PFA	298	55.3	7.21	0.231	3.98
B6CBA	F1 hybrid	PFA	855	37.5	6.6	0.0846	2.47
CBAB6	F1 hybrid	PFA	887	26.1	5.11	0.0448	1.33
CD1	Outbred	MeAc	260	78.3	8.51	0.413	6.66
CD1	Outbred	PFA	229	41.8	7.94	0.219	3.32
MF1YRIII	Outbred	MeAc	277	102	11.3	0.69	11.5
MF1YRIII	Outbred	PFA	217	37.8	7.89	0.203	2.99
LEWES	Wild-derived	PFA	551	36.5	6	0.0934	2.19
PWK	Wild-derived	PFA	543	53.1	6.84	0.156	3.63
STF	Wild-derived	PFA	583	35.2	6.19	0.0902	2.18

				**Ellipticity**			
Strain	Status	Fixative	Number of nuclei analysed	Coefficient of Variation	Mean	Standard error of the mean	Standard Deviation
BALB/c	Inbred	MeAc	420	18.4	1.2	0.0108	0.22
BALB/c	Inbred	PFA	574	16.2	1.21	0.00821	0.197
C57	Inbred	MeAc	505	11.4	1.27	0.00647	0.145
C57	Inbred	PFA	450	11.8	1.32	0.00735	0.156
CBA	Inbred	MeAc	242	9.2	1.73	0.0102	0.159
CBA	Inbred	PFA	729	10.4	1.67	0.0064	0.173
DBA	Inbred	PFA	495	11.4	1.36	0.00699	0.155
FVB	Inbred	MeAc	216	15.3	1.7	0.0177	0.26
FVB	Inbred	PFA	298	14.7	1.47	0.0124	0.215
B6CBA	F1 hybrid	PFA	855	6.58	1.6	0.0036	0.105

CBAB6	F1 hybrid	PFA	887	8.92	1.53	0.00459	0.137
CD1	Outbred	MeAc	260	15.1	1.7	0.0159	0.256
CD1	Outbred	PFA	229	10.3	1.61	0.0109	0.165
MF1YRIII	Outbred	MeAc	277	12.6	1.38	0.0104	0.173
MF1YRIII	Outbred	PFA	217	11.1	1.38	0.0105	0.154
LEWES	Wild-derived	PFA	551	9.47	1.62	0.00652	0.153
PWK	Wild-derived	PFA	543	9.64	1.79	0.0074	0.172
STF	Wild-derived	PFA	583	8.84	1.27	0.00466	0.112

				**Elongation**			
Strain	Status	Fixative	Number of nuclei analysed	Coefficient of Variation	Mean	Standard error of the mean	Standard Deviation
BALB/c	Inbred	MeAc	420	108	0.0826	0.00437	0.0895
BALB/c	Inbred	PFA	574	84.5	0.0902	0.00318	0.0762
C57	Inbred	MeAc	505	48.1	0.117	0.00251	0.0564
C57	Inbred	PFA	450	42.5	0.136	0.00272	0.0577
CBA	Inbred	MeAc	242	16.9	0.264	0.00287	0.0446
CBA	Inbred	PFA	729	20.4	0.246	0.00186	0.0503
DBA	Inbred	PFA	495	39.1	0.149	0.00261	0.0582
FVB	Inbred	MeAc	216	38.2	0.25	0.0065	0.0955
FVB	Inbred	PFA	298	36.2	0.183	0.00385	0.0664
B6CBA	F1 hybrid	PFA	855	13.7	0.23	0.00108	0.0316
CBAB6	F1 hybrid	PFA	887	22	0.208	0.00154	0.0458
CD1	Outbred	MeAc	260	29.4	0.252	0.0046	0.0742
CD1	Outbred	PFA	229	20.9	0.23	0.00317	0.048
MF1YRIII	Outbred	MeAc	277	42.1	0.154	0.00389	0.0647
MF1YRIII	Outbred	PFA	217	33	0.157	0.00352	0.0519
LEWES	Wild-derived	PFA	551	18.9	0.233	0.00187	0.044
PWK	Wild-derived	PFA	543	8.35	0.325	0.00116	0.0271
STF	Wild-derived	PFA	583	36.9	0.118	0.0018	0.0434

				**Length of hook (microns)**			
Strain	Status	Fixative	Number of nuclei analysed	Coefficient of Variation	Mean	Standard error of the mean	Standard Deviation
BALB/c	Inbred	MeAc	420	48.6	1.89	0.0448	0.917
BALB/c	Inbred	PFA	574	40.4	1.83	0.0308	0.737
C57	Inbred	MeAc	505	26.5	1.81	0.0213	0.478
C57	Inbred	PFA	450	17.7	1.9	0.0159	0.337
CBA	Inbred	MeAc	242	158	0.249	0.0253	0.393
CBA	Inbred	PFA	729	102	0.734	0.0278	0.75
DBA	Inbred	PFA	495	15.9	2.02	0.0144	0.321
FVB	Inbred	MeAc	216	143	0.31	0.0302	0.444
FVB	Inbred	PFA	298	29.1	1.52	0.0257	0.443
B6CBA	F1 hybrid	PFA	855	98.5	0.74	0.0249	0.728
CBAB6	F1 hybrid	PFA	887	17.9	1.56	0.00942	0.281
CD1	Outbred	MeAc	260	33.7	1.63	0.034	0.548
CD1	Outbred	PFA	229	20.1	1.77	0.0235	0.355
MF1YRIII	Outbred	MeAc	277	42.4	2.24	0.057	0.949
MF1YRIII	Outbred	PFA	217	16.6	2.05	0.0232	0.342
LEWES	Wild-derived	PFA	551	16.9	1.59	0.0115	0.269
PWK	Wild-derived	PFA	543	79.6	0.767	0.0262	0.611
STF	Wild-derived	PFA	583	18.2	1.55	0.0117	0.282

				**Maximum Feret distance (microns)**			
Strain	Status	Fixative	Number of nuclei analysed	Coefficient of Variation	Mean	Standard error of the mean	Standard Deviation

BALB/c	Inbred	MeAc	420	14.9	7.4	0.0537	1.1
BALB/c	Inbred	PFA	574	10.4	7.24	0.0313	0.751
C57	Inbred	MeAc	505	8.36	7.69	0.0286	0.643
C57	Inbred	PFA	450	5.08	7.69	0.0184	0.39
CBA	Inbred	MeAc	242	10.7	8.55	0.0587	0.912
CBA	Inbred	PFA	729	4.79	8.59	0.0153	0.412
DBA	Inbred	PFA	495	3.32	8.61	0.0128	0.286
FVB	Inbred	MeAc	216	14.5	7.96	0.0785	1.15
FVB	Inbred	PFA	298	3.86	7.75	0.0174	0.3
B6CBA	F1 hybrid	PFA	855	3.31	8.69	0.00983	0.287
CBAB6	F1 hybrid	PFA	887	2.92	8.66	0.00848	0.252
CD1	Outbred	MeAc	260	11	8.73	0.0594	0.958
CD1	Outbred	PFA	229	4.03	8.68	0.0231	0.35
MF1YRIII	Outbred	MeAc	277	13.6	8.93	0.0729	1.21
MF1YRIII	Outbred	PFA	217	3.09	8.57	0.018	0.265
LEWES	Wild-derived	PFA	551	3.15	8.55	0.0115	0.269
PWK	Wild-derived	PFA	543	3.36	8.79	0.0127	0.296
STF	Wild-derived	PFA	583	2.61	7.59	0.00819	0.198

				**Min diameter across centre-of-mass (microns)**			
Strain	Status	Fixative	Number of nuclei analysed	Coefficient of Variation	Mean	Standard error of the mean	Standard Deviation
BALB/c	Inbred	MeAc	420	12.7	3.28	0.0204	0.418
BALB/c	Inbred	PFA	574	9.79	3.29	0.0134	0.322
C57	Inbred	MeAc	505	6.32	3.39	0.00954	0.214
C57	Inbred	PFA	450	5.45	3.21	0.00825	0.175
CBA	Inbred	MeAc	242	7.7	3.55	0.0176	0.273
CBA	Inbred	PFA	729	4.49	3.47	0.00576	0.156
DBA	Inbred	PFA	495	3.88	3.58	0.00624	0.139
FVB	Inbred	MeAc	216	8.82	3.06	0.0184	0.27
FVB	Inbred	PFA	298	4.76	3.19	0.00878	0.152
B6CBA	F1 hybrid	PFA	855	4.07	3.38	0.0047	0.138
CBAB6	F1 hybrid	PFA	887	3.47	3.55	0.00414	0.123
CD1	Outbred	MeAc	260	8.27	3.11	0.016	0.257
CD1	Outbred	PFA	229	4.11	3.15	0.00856	0.13
MF1YRIII	Outbred	MeAc	277	11.7	3.39	0.0238	0.396
MF1YRIII	Outbred	PFA	217	5.18	3.41	0.012	0.176
LEWES	Wild-derived	PFA	551	2.9	3.22	0.00397	0.0932
PWK	Wild-derived	PFA	543	3.23	3.36	0.00466	0.109
STF	Wild-derived	PFA	583	3.33	3.5	0.00483	0.117

				**Perimeter (microns)**			
Strain	Status	Fixative	Number of nuclei analysed	Coefficient of Variation	Mean	Standard error of the mean	Standard Deviation
BALB/c	Inbred	MeAc	420	17	20.5	0.17	3.48
BALB/c	Inbred	PFA	574	8.09	19.7	0.0666	1.6
C57	Inbred	MeAc	505	5.95	20.5	0.0544	1.22
C57	Inbred	PFA	450	4.13	20.5	0.0398	0.845
CBA	Inbred	MeAc	242	10.2	21.9	0.144	2.23
CBA	Inbred	PFA	729	4.29	21.9	0.0347	0.938
DBA	Inbred	PFA	495	2.59	22.6	0.0263	0.585
FVB	Inbred	MeAc	216	17.9	20.7	0.252	3.7
FVB	Inbred	PFA	298	3.47	20	0.0403	0.695
B6CBA	F1 hybrid	PFA	855	3.1	22.4	0.0237	0.693
CBAB6	F1 hybrid	PFA	887	2.63	22.2	0.0196	0.584
CD1	Outbred	MeAc	260	13	21.8	0.176	2.84
CD1	Outbred	PFA	229	3.62	22	0.0527	0.797

MF1YRIII	Outbred	MeAc	277	20.1	23.7	0.286	4.76
MF1YRIII	Outbred	PFA	217	3	22.4	0.0457	0.674
LEWES	Wild-derived	PFA	551	2.91	21.5	0.0266	0.625
PWK	Wild-derived	PFA	543	3.18	21.8	0.0297	0.692
STF	Wild-derived	PFA	583	2.68	20.1	0.0223	0.539

				**Regularity**			
Strain	Status	Fixative	Number of nuclei analysed	Coefficient of Variation	Mean	Standard error of the mean	Standard Deviation
BALB/c	Inbred	MeAc	420	11.4	1.56	0.00872	0.179
BALB/c	Inbred	PFA	574	9.81	1.54	0.0063	0.151
C57	Inbred	MeAc	505	7.61	1.51	0.0051	0.115
C57	Inbred	PFA	450	6.1	1.57	0.00453	0.096
CBA	Inbred	MeAc	242	8.95	1.43	0.0082	0.128
CBA	Inbred	PFA	729	7.78	1.51	0.00435	0.117
DBA	Inbred	PFA	495	4.88	1.61	0.00354	0.0787
FVB	Inbred	MeAc	216	14.2	1.54	0.0148	0.218
FVB	Inbred	PFA	298	7.65	1.55	0.00687	0.119
B6CBA	F1 hybrid	PFA	855	5.42	1.56	0.0029	0.0847
CBAB6	F1 hybrid	PFA	887	5.1	1.51	0.00258	0.0767
CD1	Outbred	MeAc	260	11.2	1.65	0.0115	0.185
CD1	Outbred	PFA	229	7.07	1.66	0.00776	0.117
MF1YRIII	Outbred	MeAc	277	11	1.69	0.0112	0.186
MF1YRIII	Outbred	PFA	217	5.5	1.65	0.00617	0.0909
LEWES	Wild-derived	PFA	551	5.31	1.65	0.00373	0.0876
PWK	Wild-derived	PFA	543	5.36	1.44	0.0033	0.077
STF	Wild-derived	PFA	583	4.23	1.47	0.00257	0.0621

				**Width of body (microns)**			
Strain	Status	Fixative	Number of nuclei analysed	Coefficient of Variation	Mean	Standard error of the mean	Standard Deviation
BALB/c	Inbred	MeAc	420	17.8	3.66	0.0318	0.653
BALB/c	Inbred	PFA	574	17.4	3.56	0.0258	0.619
C57	Inbred	MeAc	505	9.76	3.62	0.0157	0.354
C57	Inbred	PFA	450	6.19	3.36	0.00981	0.208
CBA	Inbred	MeAc	242	11.5	4.49	0.0333	0.518
CBA	Inbred	PFA	729	14	4.14	0.0215	0.579
DBA	Inbred	PFA	495	4.97	3.73	0.00834	0.186
FVB	Inbred	MeAc	216	21.4	4.18	0.0607	0.892
FVB	Inbred	PFA	298	9.75	3.36	0.019	0.328
B6CBA	F1 hybrid	PFA	855	16.4	4.36	0.0245	0.716
CBAB6	F1 hybrid	PFA	887	4.06	3.65	0.00498	0.148
CD1	Outbred	MeAc	260	15.5	3.23	0.0311	0.501
CD1	Outbred	PFA	229	4.94	3.25	0.0106	0.16
MF1YRIII	Outbred	MeAc	277	21.8	3.65	0.0478	0.796
MF1YRIII	Outbred	PFA	217	5.3	3.57	0.0128	0.189
LEWES	Wild-derived	PFA	551	3.87	3.31	0.00547	0.128
PWK	Wild-derived	PFA	543	10.7	3.88	0.0177	0.414
STF	Wild-derived	PFA	583	4.25	3.74	0.00657	0.159
