# Supplementary material for: A high-throughput method for unbiased quantitation and categorization of nuclear morphology
Source: Biol Reprod. 2019 Feb 11;100(5):1250–60. doi: 10.1093/biolre/ioz013 (PMC6497523; doi:10.1093/biolre/ioz013)
Supplement: ioz013_Supplemental_Files [file ioz013_supplemental_files.zip › Supplementary table 2.pdf]

Supplementary Table 2: Parameters measured by the software for each sample

						Angle between reference points (degrees)			
Status	Strain	Sample	Fixative	Number of animals pooled	Number of nuclei analysed	Coefficient of Variation	Mean	Standard error of the mean	Standard Deviation
Inbred	Balbc	Balbc 1	MeAc	1	201	6.62	142	0.661	9.38
Inbred	Balbc	Balbc 1	PFA	1	281	5.37	140	0.449	7.53
Inbred	Balbc	Balbc 2	MeAc	1	219	9.85	142	0.942	13.9
Inbred	Balbc	Balbc 2	PFA	1	293	5.74	142	0.475	8.14
Inbred	C57	C57 3	MeAc	1	253	3.07	139	0.269	4.28
Inbred	C57	C57 3	PFA	1	213	1.97	138	0.187	2.73
Inbred	C57	C57 4	MeAc	1	252	2.99	141	0.265	4.21
Inbred	C57	C57 4	PFA	1	237	1.9	139	0.171	2.64
Inbred	CBA	CBA 1	PFA	1	177	1.61	146	0.178	2.36
Inbred	CBA	CBA 2	PFA	1	308	1.41	146	0.117	2.06
Inbred	CBA	CBA 3	MeAc	1	242	7.08	148	0.673	10.5
Inbred	CBA	CBA 3	PFA	1	244	1.88	147	0.177	2.76
Inbred	DBA	DBA 1	PFA	1	244	2.06	139	0.183	2.86
Inbred	DBA	DBA 2	PFA	1	251	1.91	139	0.167	2.65
Inbred	FVB	FVB 1	MeAc	1	216	7.91	149	0.802	11.8
Inbred	FVB	FVB 1	PFA	1	298	2.55	148	0.218	3.76
F1 hybrid	B6CBA	B6CBA 1	PFA	1	324	1.41	143	0.112	2.01
F1 hybrid	B6CBA	B6CBA 2	PFA	1	313	1.56	142	0.125	2.22
F1 hybrid	B6CBA	B6CBA 4	PFA	1	218	1.69	143	0.164	2.41
F1 hybrid	CBAB6	CBAB6 1	PFA	1	217	1.36	143	0.132	1.95
F1 hybrid	CBAB6	CBAB6 2	PFA	1	215	1.24	142	0.121	1.77
F1 hybrid	CBAB6	CBAB6 3	PFA	1	232	1.22	143	0.115	1.75
F1 hybrid	CBAB6	CBAB6 4	PFA	1	223	1.17	144	0.112	1.67
Outbred	CD1	CD1 1	MeAc	15	260	3.08	147	0.281	4.54
Outbred	CD1	CD1 1	PFA	15	229	2.2	143	0.208	3.15
Outbred	MF1YRIII	MF1YRIII 2	MeAc	8	277	7.09	141	0.599	9.97
Outbred	MF1YRIII	MF1YRIII 2	PFA	8	217	2.19	139	0.207	3.05
Wild-derived	LEWES	LEWES 1	PFA	2	273	1.45	147	0.129	2.14
Wild-derived	LEWES	LEWES 2	PFA	2	278	1.3	147	0.114	1.91
Wild-derived	PWK	PWK 2	PFA	2	275	1.41	153	0.13	2.15
Wild-derived	PWK	PWK 3	PFA	2	268	1.61	151	0.149	2.43
Wild-derived	STF	STF 1	PFA	2	288	2.43	136	0.196	3.32
Wild-derived	STF	STF 2	PFA	2	295	1.97	138	0.158	2.71

						Area (square microns)			
Status	Strain	Sample	Fixative	Number of animals pooled	Number of nuclei analysed	Coefficient of Variation	Mean	Standard error of the mean	Standard Deviation
Inbred	Balbc	Balbc 1	MeAc	1	201	22.5	18.5	0.294	4.16
Inbred	Balbc	Balbc 1	PFA	1	281	12.5	17.6	0.131	2.19
Inbred	Balbc	Balbc 2	MeAc	1	219	20.5	17.6	0.244	3.61
Inbred	Balbc	Balbc 2	PFA	1	293	11.6	17.4	0.117	2.01
Inbred	C57	C57 3	MeAc	1	253	6.56	19.1	0.079	1.26
Inbred	C57	C57 3	PFA	1	213	5.95	17.8	0.0728	1.06
Inbred	C57	C57 4	MeAc	1	252	7.75	19.3	0.0943	1.5
Inbred	C57	C57 4	PFA	1	237	5.74	18	0.0673	1.04
Inbred	CBA	CBA 1	PFA	1	177	6.15	20.5	0.0947	1.26
Inbred	CBA	CBA 2	PFA	1	308	5.87	20.3	0.068	1.19
Inbred	CBA	CBA 3	MeAc	1	242	12.6	21	0.171	2.65
Inbred	CBA	CBA 3	PFA	1	244	5.74	19.7	0.0726	1.13
Inbred	DBA	DBA 1	PFA	1	244	4.68	21.3	0.0639	0.998
Inbred	DBA	DBA 2	PFA	1	251	3.54	21.6	0.0484	0.767

Inbred	FVB	FVB 1	MeAc	1	216	20.9	16.8	0.239	3.51
Inbred	FVB	FVB 1	PFA	1	298	5.53	17.2	0.055	0.949
F1 hybrid	B6CBA	B6CBA 1	PFA	1	324	4.73	20.6	0.0541	0.974
F1 hybrid	B6CBA	B6CBA 2	PFA	1	313	4.94	20.5	0.0572	1.01
F1 hybrid	B6CBA	B6CBA 4	PFA	1	218	4.58	20.6	0.0639	0.943
F1 hybrid	CBAB6	CBAB6 1	PFA	1	217	4.28	20.2	0.0588	0.867
F1 hybrid	CBAB6	CBAB6 2	PFA	1	215	3.74	21.2	0.0541	0.794
F1 hybrid	CBAB6	CBAB6 3	PFA	1	232	3.54	21.7	0.0505	0.77
F1 hybrid	CBAB6	CBAB6 4	PFA	1	223	3.49	22.2	0.0518	0.774
Outbred	CD1	CD1 1	MeAc	15	260	15.3	18.5	0.175	2.82
Outbred	CD1	CD1 1	PFA	15	229	4.81	18.8	0.0596	0.902
Outbred	MF1YRIII	MF1YRIII 2	MeAc	8	277	23.6	21.8	0.309	5.14
Outbred	MF1YRIII	MF1YRIII 2	PFA	8	217	5.17	20.3	0.0713	1.05
Wild-derived	LEWES	LEWES 1	PFA	2	273	4	18.2	0.0441	0.728
Wild-derived	LEWES	LEWES 2	PFA	2	278	3.78	18.1	0.041	0.683
Wild-derived	PWK	PWK 2	PFA	2	275	4.36	19.7	0.0518	0.858
Wild-derived	PWK	PWK 3	PFA	2	268	5.26	19.3	0.062	1.02
Wild-derived	STF	STF 1	PFA	2	288	4.48	18.7	0.0493	0.837
Wild-derived	STF	STF 2	PFA	2	295	3.63	18.6	0.0393	0.675

						**Aspect ratio**			
Status	Strain	Sample	Fixative	Number of animals pooled	Number of nuclei analysed	Coefficient of Variation	Mean	Standard error of the mean	Standard Deviation
Inbred	Balbc	Balbc 1	MeAc	1	201	17.9	0.864	0.0109	0.154
Inbred	Balbc	Balbc 1	PFA	1	281	14.3	0.854	0.00729	0.122
Inbred	Balbc	Balbc 2	MeAc	1	219	20.7	0.858	0.012	0.178
Inbred	Balbc	Balbc 2	PFA	1	293	16.6	0.833	0.00806	0.138
Inbred	C57	C57 3	MeAc	1	253	11.2	0.804	0.00564	0.0896
Inbred	C57	C57 3	PFA	1	213	11.8	0.781	0.00629	0.0918
Inbred	C57	C57 4	MeAc	1	252	12.6	0.786	0.00624	0.0991
Inbred	C57	C57 4	PFA	1	237	12.2	0.753	0.00597	0.092
Inbred	CBA	CBA 1	PFA	1	177	5.03	0.583	0.00221	0.0293
Inbred	CBA	CBA 2	PFA	1	308	14.2	0.639	0.00518	0.091
Inbred	CBA	CBA 3	MeAc	1	242	10.6	0.585	0.00398	0.0618
Inbred	CBA	CBA 3	PFA	1	244	5.6	0.585	0.0021	0.0327
Inbred	DBA	DBA 1	PFA	1	244	15.3	0.751	0.00737	0.115
Inbred	DBA	DBA 2	PFA	1	251	13.3	0.742	0.00625	0.0989
Inbred	FVB	FVB 1	MeAc	1	216	31.6	0.614	0.0132	0.194
Inbred	FVB	FVB 1	PFA	1	298	16.3	0.696	0.00656	0.113
F1 hybrid	B6CBA	B6CBA 1	PFA	1	324	4.17	0.615	0.00143	0.0257
F1 hybrid	B6CBA	B6CBA 2	PFA	1	313	9.02	0.639	0.00326	0.0577
F1 hybrid	B6CBA	B6CBA 4	PFA	1	218	5	0.626	0.00212	0.0313
F1 hybrid	CBAB6	CBAB6 1	PFA	1	217	13.2	0.667	0.00596	0.0877
F1 hybrid	CBAB6	CBAB6 2	PFA	1	215	9.63	0.652	0.00428	0.0628
F1 hybrid	CBAB6	CBAB6 3	PFA	1	232	15.4	0.659	0.00667	0.102
F1 hybrid	CBAB6	CBAB6 4	PFA	1	223	7.1	0.654	0.00311	0.0464
Outbred	CD1	CD1 1	MeAc	15	260	17.8	0.603	0.00666	0.107
Outbred	CD1	CD1 1	PFA	15	229	10.1	0.629	0.00422	0.0638
Outbred	MF1YRIII	MF1YRIII 2	MeAc	8	277	15	0.74	0.00666	0.111
Outbred	MF1YRIII	MF1YRIII 2	PFA	8	217	10.6	0.731	0.00526	0.0775
Wild-derived	LEWES	LEWES 1	PFA	2	273	9.05	0.62	0.0034	0.0561
Wild-derived	LEWES	LEWES 2	PFA	2	278	9.6	0.629	0.00362	0.0604
Wild-derived	PWK	PWK 2	PFA	2	275	4.68	0.494	0.00139	0.0231
Wild-derived	PWK	PWK 3	PFA	2	268	5.71	0.527	0.00184	0.0301
Wild-derived	STF	STF 1	PFA	2	288	9.48	0.792	0.00443	0.0751
Wild-derived	STF	STF 2	PFA	2	295	8.4	0.792	0.00387	0.0665

						Bounding height (microns)			
Status	Strain	Sample	Fixative	Number of animals pooled	Number of nuclei analysed	Coefficient of Variation	Mean	Standard error of the mean	Standard Deviation
Inbred	Balbc	Balbc 1	MeAc	1	201	15.8	6.54	0.073	1.04
Inbred	Balbc	Balbc 1	PFA	1	281	10.1	6.39	0.0386	0.647
Inbred	Balbc	Balbc 2	MeAc	1	219	14.3	6.45	0.0625	0.924
Inbred	Balbc	Balbc 2	PFA	1	293	9.69	6.41	0.0363	0.621
Inbred	C57	C57 3	MeAc	1	253	8.13	6.74	0.0344	0.548
Inbred	C57	C57 3	PFA	1	213	7.02	6.79	0.0327	0.477
Inbred	C57	C57 4	MeAc	1	252	8.69	6.94	0.038	0.604
Inbred	C57	C57 4	PFA	1	237	5.97	6.95	0.027	0.415
Inbred	CBA	CBA 1	PFA	1	177	3.46	8.2	0.0213	0.284
Inbred	CBA	CBA 2	PFA	1	308	5.57	7.99	0.0254	0.445
Inbred	CBA	CBA 3	MeAc	1	242	10.5	8.09	0.0545	0.847
Inbred	CBA	CBA 3	PFA	1	244	4.73	7.87	0.0238	0.372
Inbred	DBA	DBA 1	PFA	1	244	5.96	7.67	0.0293	0.457
Inbred	DBA	DBA 2	PFA	1	251	5.06	7.78	0.0249	0.394
Inbred	FVB	FVB 1	MeAc	1	216	15.8	7.4	0.0795	1.17
Inbred	FVB	FVB 1	PFA	1	298	5.28	7	0.0214	0.37
F1 hybrid	B6CBA	B6CBA 1	PFA	1	324	2.58	8.25	0.0118	0.213
F1 hybrid	B6CBA	B6CBA 2	PFA	1	313	4.02	8	0.0182	0.321
F1 hybrid	B6CBA	B6CBA 4	PFA	1	218	2.9	7.98	0.0157	0.231
F1 hybrid	CBAB6	CBAB6 1	PFA	1	217	4.52	7.77	0.0239	0.352
F1 hybrid	CBAB6	CBAB6 2	PFA	1	215	4.02	7.79	0.0214	0.313
F1 hybrid	CBAB6	CBAB6 3	PFA	1	232	4.29	7.98	0.0225	0.342
F1 hybrid	CBAB6	CBAB6 4	PFA	1	223	2.85	8.06	0.0154	0.23
Outbred	CD1	CD1 1	MeAc	15	260	10.8	8.04	0.0537	0.866
Outbred	CD1	CD1 1	PFA	15	229	4.53	7.95	0.0238	0.361
Outbred	MF1YRIII	MF1YRIII 2	MeAc	8	277	13.5	7.95	0.0643	1.07
Outbred	MF1YRIII	MF1YRIII 2	PFA	8	217	4.17	7.66	0.0217	0.32
Wild-derived	LEWES	LEWES 1	PFA	2	273	3.94	7.86	0.0188	0.31
Wild-derived	LEWES	LEWES 2	PFA	2	278	4.12	7.79	0.0192	0.321
Wild-derived	PWK	PWK 2	PFA	2	275	3.49	8.09	0.017	0.282
Wild-derived	PWK	PWK 3	PFA	2	268	3.82	8.29	0.0194	0.317
Wild-derived	STF	STF 1	PFA	2	288	4.09	6.64	0.016	0.272
Wild-derived	STF	STF 2	PFA	2	295	3.51	6.64	0.0136	0.233

						Bounding width (microns)			
Status	Strain	Sample	Fixative	Number of animals pooled	Number of nuclei analysed	Coefficient of Variation	Mean	Standard error of the mean	Standard Deviation
Inbred	Balbc	Balbc 1	MeAc	1	201	16	5.57	0.0626	0.888
Inbred	Balbc	Balbc 1	PFA	1	281	11.7	5.42	0.0378	0.634
Inbred	Balbc	Balbc 2	MeAc	1	219	17.6	5.46	0.0647	0.958
Inbred	Balbc	Balbc 2	PFA	1	293	12.7	5.29	0.0393	0.673
Inbred	C57	C57 3	MeAc	1	253	7.41	5.38	0.0251	0.399
Inbred	C57	C57 3	PFA	1	213	7.09	5.26	0.0256	0.373
Inbred	C57	C57 4	MeAc	1	252	7.61	5.41	0.0259	0.412
Inbred	C57	C57 4	PFA	1	237	7.92	5.2	0.0268	0.412
Inbred	CBA	CBA 1	PFA	1	177	6.08	4.78	0.0218	0.291
Inbred	CBA	CBA 2	PFA	1	308	9.76	5.07	0.0282	0.495
Inbred	CBA	CBA 3	MeAc	1	242	9.23	4.7	0.0279	0.434
Inbred	CBA	CBA 3	PFA	1	244	6.9	4.6	0.0203	0.318
Inbred	DBA	DBA 1	PFA	1	244	7.56	5.71	0.0276	0.432
Inbred	DBA	DBA 2	PFA	1	251	6.98	5.74	0.0253	0.401

Inbred	FVB	FVB 1	MeAc	1	216	21.1	4.45	0.0638	0.937
Inbred	FVB	FVB 1	PFA	1	298	10.2	4.85	0.0286	0.494
F1 hybrid	B6CBA	B6CBA 1	PFA	1	324	4.48	5.08	0.0126	0.227
F1 hybrid	B6CBA	B6CBA 2	PFA	1	313	6.62	5.1	0.0191	0.337
F1 hybrid	B6CBA	B6CBA 4	PFA	1	218	5.35	4.99	0.0181	0.267
F1 hybrid	CBAB6	CBAB6 1	PFA	1	217	8.15	5.16	0.0285	0.42
F1 hybrid	CBAB6	CBAB6 2	PFA	1	215	6.65	5.07	0.023	0.337
F1 hybrid	CBAB6	CBAB6 3	PFA	1	232	6.29	5.23	0.0216	0.329
F1 hybrid	CBAB6	CBAB6 4	PFA	1	223	5.23	5.27	0.0184	0.275
Outbred	CD1	CD1 1	MeAc	15	260	17.9	4.82	0.0536	0.865
Outbred	CD1	CD1 1	PFA	15	229	7.92	4.99	0.0261	0.395
Outbred	MF1YRIII	MF1YRIII 2	MeAc	8	277	18.2	5.86	0.0641	1.07
Outbred	MF1YRIII	MF1YRIII 2	PFA	8	217	7.61	5.58	0.0288	0.425
Wild-derived	LEWES	LEWES 1	PFA	2	273	6.99	4.86	0.0206	0.34
Wild-derived	LEWES	LEWES 2	PFA	2	278	6.92	4.89	0.0203	0.338
Wild-derived	PWK	PWK 2	PFA	2	275	6.78	4.86	0.0199	0.33
Wild-derived	PWK	PWK 3	PFA	2	268	5.06	4.37	0.0135	0.221
Wild-derived	STF	STF 1	PFA	2	288	6.49	5.25	0.0201	0.341
Wild-derived	STF	STF 2	PFA	2	295	5.88	5.25	0.018	0.309

						**Circularity**			
Status	Strain	Sample	Fixative	Number of animals pooled	Number of nuclei analysed	Coefficient of Variation	Mean	Standard error of the mean	Standard Deviation
Inbred	Balbc	Balbc 1	MeAc	1	201	13.7	0.554	0.00536	0.076
Inbred	Balbc	Balbc 1	PFA	1	281	9.35	0.565	0.00315	0.0528
Inbred	Balbc	Balbc 2	MeAc	1	219	13.6	0.544	0.00499	0.0738
Inbred	Balbc	Balbc 2	PFA	1	293	9.93	0.567	0.00329	0.0563
Inbred	C57	C57 3	MeAc	1	253	7.56	0.586	0.00278	0.0443
Inbred	C57	C57 3	PFA	1	213	5.88	0.542	0.00218	0.0318
Inbred	C57	C57 4	MeAc	1	252	8.47	0.564	0.00301	0.0478
Inbred	C57	C57 4	PFA	1	237	5.67	0.537	0.00198	0.0305
Inbred	CBA	CBA 1	PFA	1	177	4.44	0.523	0.00175	0.0232
Inbred	CBA	CBA 2	PFA	1	308	5.45	0.52	0.00162	0.0284
Inbred	CBA	CBA 3	MeAc	1	242	9.88	0.555	0.00352	0.0548
Inbred	CBA	CBA 3	PFA	1	244	7.1	0.547	0.00249	0.0388
Inbred	DBA	DBA 1	PFA	1	244	4.18	0.531	0.00142	0.0222
Inbred	DBA	DBA 2	PFA	1	251	3.87	0.529	0.00129	0.0204
Inbred	FVB	FVB 1	MeAc	1	216	14.3	0.502	0.0049	0.072
Inbred	FVB	FVB 1	PFA	1	298	6.04	0.539	0.00189	0.0326
F1 hybrid	B6CBA	B6CBA 1	PFA	1	324	4.06	0.502	0.00113	0.0204
F1 hybrid	B6CBA	B6CBA 2	PFA	1	313	4.35	0.521	0.00128	0.0227
F1 hybrid	B6CBA	B6CBA 4	PFA	1	218	5.25	0.532	0.00189	0.0279
F1 hybrid	CBAB6	CBAB6 1	PFA	1	217	3.53	0.529	0.00127	0.0187
F1 hybrid	CBAB6	CBAB6 2	PFA	1	215	3.37	0.561	0.00129	0.0189
F1 hybrid	CBAB6	CBAB6 3	PFA	1	232	3.02	0.544	0.00108	0.0165
F1 hybrid	CBAB6	CBAB6 4	PFA	1	223	2.93	0.547	0.00107	0.016
Outbred	CD1	CD1 1	MeAc	15	260	9.35	0.492	0.00286	0.046
Outbred	CD1	CD1 1	PFA	15	229	6.42	0.487	0.00207	0.0313
Outbred	MF1YRIII	MF1YRIII 2	MeAc	8	277	14.4	0.498	0.00431	0.0717
Outbred	MF1YRIII	MF1YRIII 2	PFA	8	217	4.78	0.507	0.00164	0.0242
Wild-derived	LEWES	LEWES 1	PFA	2	273	4.23	0.494	0.00127	0.0209
Wild-derived	LEWES	LEWES 2	PFA	2	278	3.98	0.495	0.00118	0.0197
Wild-derived	PWK	PWK 2	PFA	2	275	3.99	0.519	0.00125	0.0207
Wild-derived	PWK	PWK 3	PFA	2	268	5.11	0.516	0.00161	0.0264
Wild-derived	STF	STF 1	PFA	2	288	4.24	0.578	0.00144	0.0245
Wild-derived	STF	STF 2	PFA	2	295	3.56	0.577	0.0012	0.0206

						Variability			
Status	Strain	Sample	Fixative	Number of animals pooled	Number of nuclei analysed	Coefficient of Variation	Mean	Standard error of the mean	Standard Deviation
Inbred	Balbc	Balbc 1	MeAc	1	201	60.7	16.1	0.69	9.78
Inbred	Balbc	Balbc 1	PFA	1	281	46.8	14.6	0.407	6.83
Inbred	Balbc	Balbc 2	MeAc	1	219	61.9	17.5	0.732	10.8
Inbred	Balbc	Balbc 2	PFA	1	293	46.6	14.9	0.405	6.93
Inbred	C57	C57 3	MeAc	1	253	74.5	8.97	0.42	6.68
Inbred	C57	C57 3	PFA	1	213	39.3	8.54	0.23	3.36
Inbred	C57	C57 4	MeAc	1	252	70.9	9.46	0.422	6.71
Inbred	C57	C57 4	PFA	1	237	40.5	8.18	0.215	3.31
Inbred	CBA	CBA 1	PFA	1	177	38.3	6.61	0.19	2.53
Inbred	CBA	CBA 2	PFA	1	308	42.4	7.54	0.182	3.2
Inbred	CBA	CBA 3	MeAc	1	242	72.3	9.16	0.426	6.62
Inbred	CBA	CBA 3	PFA	1	244	43.1	8.27	0.228	3.57
Inbred	DBA	DBA 1	PFA	1	244	34.2	7.07	0.154	2.41
Inbred	DBA	DBA 2	PFA	1	251	32.6	6.44	0.133	2.1
Inbred	FVB	FVB 1	MeAc	1	216	81	15.6	0.86	12.6
Inbred	FVB	FVB 1	PFA	1	298	55.3	7.21	0.231	3.98
F1 hybrid	B6CBA	B6CBA 1	PFA	1	324	38.7	6.53	0.14	2.53
F1 hybrid	B6CBA	B6CBA 2	PFA	1	313	38	6.58	0.141	2.5
F1 hybrid	B6CBA	B6CBA 4	PFA	1	218	35.2	6.72	0.16	2.37
F1 hybrid	CBAB6	CBAB6 1	PFA	1	217	25.1	5.32	0.0909	1.34
F1 hybrid	CBAB6	CBAB6 2	PFA	1	215	25.1	5.33	0.0914	1.34
F1 hybrid	CBAB6	CBAB6 3	PFA	1	232	26.9	4.88	0.0863	1.31
F1 hybrid	CBAB6	CBAB6 4	PFA	1	223	26.1	4.92	0.0861	1.29
Outbred	CD1	CD1 1	MeAc	15	260	78.3	8.51	0.413	6.66
Outbred	CD1	CD1 1	PFA	15	229	41.8	7.94	0.219	3.32
Outbred	MF1YRIII	MF1YRIII 2	MeAc	8	277	102	11.3	0.69	11.5
Outbred	MF1YRIII	MF1YRIII 2	PFA	8	217	37.8	7.89	0.203	2.99
Wild-derived	LEWES	LEWES 1	PFA	2	273	34.3	5.97	0.124	2.05
Wild-derived	LEWES	LEWES 2	PFA	2	278	38.6	6.03	0.14	2.33
Wild-derived	PWK	PWK 2	PFA	2	275	52.5	6.62	0.21	3.48
Wild-derived	PWK	PWK 3	PFA	2	268	53.4	7.07	0.231	3.78
Wild-derived	STF	STF 1	PFA	2	288	35.4	6.32	0.132	2.24
Wild-derived	STF	STF 2	PFA	2	295	34.9	6.05	0.123	2.11

						Ellipticity			
Status	Strain	Sample	Fixative	Number of animals pooled	Number of nuclei analysed	Coefficient of Variation	Mean	Standard error of the mean	Standard Deviation
Inbred	Balbc	Balbc 1	MeAc	1	201	18.9	1.2	0.0159	0.226
Inbred	Balbc	Balbc 1	PFA	1	281	15.4	1.2	0.011	0.184
Inbred	Balbc	Balbc 2	MeAc	1	219	17.9	1.21	0.0146	0.216
Inbred	Balbc	Balbc 2	PFA	1	293	16.8	1.23	0.0121	0.207
Inbred	C57	C57 3	MeAc	1	253	11.5	1.26	0.00914	0.145
Inbred	C57	C57 3	PFA	1	213	11.6	1.3	0.0103	0.15
Inbred	C57	C57 4	MeAc	1	252	11.2	1.29	0.00908	0.144
Inbred	C57	C57 4	PFA	1	237	11.7	1.35	0.0102	0.157
Inbred	CBA	CBA 1	PFA	1	177	5.25	1.72	0.00679	0.0903
Inbred	CBA	CBA 2	PFA	1	308	14	1.59	0.0127	0.223
Inbred	CBA	CBA 3	MeAc	1	242	9.2	1.73	0.0102	0.159
Inbred	CBA	CBA 3	PFA	1	244	5.86	1.72	0.00643	0.1
Inbred	DBA	DBA 1	PFA	1	244	12.1	1.35	0.0105	0.164
Inbred	DBA	DBA 2	PFA	1	251	10.8	1.37	0.00928	0.147

Inbred	FVB	FVB 1	MeAc	1	216	15.3	1.7	0.0177	0.26
Inbred	FVB	FVB 1	PFA	1	298	14.7	1.47	0.0124	0.215
F1 hybrid	B6CBA	B6CBA 1	PFA	1	324	4.27	1.63	0.00386	0.0694
F1 hybrid	B6CBA	B6CBA 2	PFA	1	313	8.8	1.58	0.00784	0.139
F1 hybrid	B6CBA	B6CBA 4	PFA	1	218	5.18	1.6	0.00562	0.083
F1 hybrid	CBAB6	CBAB6 1	PFA	1	217	11.2	1.52	0.0116	0.171
F1 hybrid	CBAB6	CBAB6 2	PFA	1	215	9.52	1.55	0.01	0.147
F1 hybrid	CBAB6	CBAB6 3	PFA	1	232	7.64	1.53	0.00768	0.117
F1 hybrid	CBAB6	CBAB6 4	PFA	1	223	6.77	1.54	0.00697	0.104
Outbred	CD1	CD1 1	MeAc	15	260	15.1	1.7	0.0159	0.256
Outbred	CD1	CD1 1	PFA	15	229	10.3	1.61	0.0109	0.165
Outbred	MF1YRIII	MF1YRIII 2	MeAc	8	277	12.6	1.38	0.0104	0.173
Outbred	MF1YRIII	MF1YRIII 2	PFA	8	217	11.1	1.38	0.0105	0.154
Wild-derived	LEWES	LEWES 1	PFA	2	273	9.47	1.63	0.00933	0.154
Wild-derived	LEWES	LEWES 2	PFA	2	278	9.42	1.6	0.00906	0.151
Wild-derived	PWK	PWK 2	PFA	2	275	8.96	1.67	0.00905	0.15
Wild-derived	PWK	PWK 3	PFA	2	268	5.38	1.9	0.00626	0.102
Wild-derived	STF	STF 1	PFA	2	288	9.35	1.27	0.00701	0.119
Wild-derived	STF	STF 2	PFA	2	295	8.33	1.27	0.00616	0.106

						**Elongation**			
Status	Strain	Sample	Fixative	Number of animals pooled	Number of nuclei analysed	Coefficient of Variation	Mean	Standard error of the mean	Standard Deviation
Inbred	Balbc	Balbc 1	MeAc	1	201	111	0.08	0.00624	0.0885
Inbred	Balbc	Balbc 1	PFA	1	281	86.4	0.0833	0.00429	0.072
Inbred	Balbc	Balbc 2	MeAc	1	219	107	0.085	0.00612	0.0906
Inbred	Balbc	Balbc 2	PFA	1	293	82.2	0.0967	0.00465	0.0795
Inbred	C57	C57 3	MeAc	1	253	48.9	0.112	0.00343	0.0546
Inbred	C57	C57 3	PFA	1	213	45	0.126	0.00389	0.0568
Inbred	C57	C57 4	MeAc	1	252	46.9	0.123	0.00363	0.0577
Inbred	C57	C57 4	PFA	1	237	39.8	0.144	0.00372	0.0573
Inbred	CBA	CBA 1	PFA	1	177	8.99	0.264	0.00178	0.0237
Inbred	CBA	CBA 2	PFA	1	308	29	0.224	0.0037	0.065
Inbred	CBA	CBA 3	MeAc	1	242	16.9	0.264	0.00287	0.0446
Inbred	CBA	CBA 3	PFA	1	244	10.1	0.262	0.00169	0.0264
Inbred	DBA	DBA 1	PFA	1	244	42.1	0.146	0.00394	0.0615
Inbred	DBA	DBA 2	PFA	1	251	36.2	0.151	0.00345	0.0547
Inbred	FVB	FVB 1	MeAc	1	216	38.2	0.25	0.0065	0.0955
Inbred	FVB	FVB 1	PFA	1	298	36.2	0.183	0.00385	0.0664
F1 hybrid	B6CBA	B6CBA 1	PFA	1	324	8.31	0.238	0.0011	0.0198
F1 hybrid	B6CBA	B6CBA 2	PFA	1	313	19	0.222	0.00238	0.0421
F1 hybrid	B6CBA	B6CBA 4	PFA	1	218	10.4	0.23	0.00162	0.0239
F1 hybrid	CBAB6	CBAB6 1	PFA	1	217	27.2	0.203	0.00375	0.0552
F1 hybrid	CBAB6	CBAB6 2	PFA	1	215	21.4	0.212	0.00309	0.0453
F1 hybrid	CBAB6	CBAB6 3	PFA	1	232	22.6	0.208	0.00309	0.0471
F1 hybrid	CBAB6	CBAB6 4	PFA	1	223	15.8	0.21	0.00222	0.0331
Outbred	CD1	CD1 1	MeAc	15	260	29.4	0.252	0.0046	0.0742
Outbred	CD1	CD1 1	PFA	15	229	20.9	0.23	0.00317	0.048
Outbred	MF1YRIII	MF1YRIII 2	MeAc	8	277	42.1	0.154	0.00389	0.0647
Outbred	MF1YRIII	MF1YRIII 2	PFA	8	217	33	0.157	0.00352	0.0519
Wild-derived	LEWES	LEWES 1	PFA	2	273	18.3	0.236	0.00261	0.0431
Wild-derived	LEWES	LEWES 2	PFA	2	278	19.5	0.229	0.00268	0.0447
Wild-derived	PWK	PWK 2	PFA	2	275	6.13	0.339	0.00125	0.0208
Wild-derived	PWK	PWK 3	PFA	2	268	8.02	0.31	0.00152	0.0249
Wild-derived	STF	STF 1	PFA	2	288	39	0.118	0.0027	0.0459
Wild-derived	STF	STF 2	PFA	2	295	34.7	0.118	0.00238	0.0409

						Length of hook (microns)			
Status	Strain	Sample	Fixative	Number of animals pooled	Number of nuclei analysed	Coefficient of Variation	Mean	Standard error of the mean	Standard Deviation
Inbred	Balbc	Balbc 1	MeAc	1	201	50.2	1.88	0.0664	0.941
Inbred	Balbc	Balbc 1	PFA	1	281	39.8	1.86	0.0442	0.742
Inbred	Balbc	Balbc 2	MeAc	1	219	47.3	1.9	0.0606	0.897
Inbred	Balbc	Balbc 2	PFA	1	293	40.9	1.79	0.0428	0.733
Inbred	C57	C57 3	MeAc	1	253	25	1.76	0.0277	0.44
Inbred	C57	C57 3	PFA	1	213	16.5	1.9	0.0214	0.312
Inbred	C57	C57 4	MeAc	1	252	27.6	1.85	0.0322	0.511
Inbred	C57	C57 4	PFA	1	237	18.8	1.91	0.0233	0.358
Inbred	CBA	CBA 1	PFA	1	177	44.7	0.102	0.00343	0.0456
Inbred	CBA	CBA 2	PFA	1	308	27.1	1.55	0.0239	0.42
Inbred	CBA	CBA 3	MeAc	1	242	158	0.249	0.0253	0.393
Inbred	CBA	CBA 3	PFA	1	244	43.9	0.165	0.00463	0.0723
Inbred	DBA	DBA 1	PFA	1	244	16.5	2.02	0.0213	0.332
Inbred	DBA	DBA 2	PFA	1	251	15.4	2.02	0.0197	0.311
Inbred	FVB	FVB 1	MeAc	1	216	143	0.31	0.0302	0.444
Inbred	FVB	FVB 1	PFA	1	298	29.1	1.52	0.0257	0.443
F1 hybrid	B6CBA	B6CBA 1	PFA	1	324	28.8	0.201	0.00322	0.0579
F1 hybrid	B6CBA	B6CBA 2	PFA	1	313	17.5	1.67	0.0165	0.291
F1 hybrid	B6CBA	B6CBA 4	PFA	1	218	27.9	0.208	0.00394	0.0582
F1 hybrid	CBAB6	CBAB6 1	PFA	1	217	19.8	1.64	0.022	0.324
F1 hybrid	CBAB6	CBAB6 2	PFA	1	215	19.6	1.45	0.0193	0.284
F1 hybrid	CBAB6	CBAB6 3	PFA	1	232	15.7	1.58	0.0162	0.247
F1 hybrid	CBAB6	CBAB6 4	PFA	1	223	14.2	1.6	0.0151	0.226
Outbred	CD1	CD1 1	MeAc	15	260	33.7	1.63	0.034	0.548
Outbred	CD1	CD1 1	PFA	15	229	20.1	1.77	0.0235	0.355
Outbred	MF1YRIII	MF1YRIII 2	MeAc	8	277	42.4	2.24	0.057	0.949
Outbred	MF1YRIII	MF1YRIII 2	PFA	8	217	16.6	2.05	0.0232	0.342
Wild-derived	LEWES	LEWES 1	PFA	2	273	18	1.58	0.0172	0.285
Wild-derived	LEWES	LEWES 2	PFA	2	278	15.8	1.6	0.0152	0.253
Wild-derived	PWK	PWK 2	PFA	2	275	20.7	1.33	0.0167	0.276
Wild-derived	PWK	PWK 3	PFA	2	268	59.5	0.188	0.00683	0.112
Wild-derived	STF	STF 1	PFA	2	288	19.7	1.54	0.0178	0.302
Wild-derived	STF	STF 2	PFA	2	295	16.8	1.55	0.0151	0.26

						Maximum Feret distance (microns)			
Status	Strain	Sample	Fixative	Number of animals pooled	Number of nuclei analysed	Coefficient of Variation	Mean	Standard error of the mean	Standard Deviation
Inbred	Balbc	Balbc 1	MeAc	1	201	14.9	7.47	0.0786	1.11
Inbred	Balbc	Balbc 1	PFA	1	281	10.7	7.27	0.0463	0.777
Inbred	Balbc	Balbc 2	MeAc	1	219	14.8	7.34	0.0735	1.09
Inbred	Balbc	Balbc 2	PFA	1	293	10	7.21	0.0423	0.725
Inbred	C57	C57 3	MeAc	1	253	8.19	7.61	0.0391	0.623
Inbred	C57	C57 3	PFA	1	213	5.49	7.64	0.0287	0.419
Inbred	C57	C57 4	MeAc	1	252	8.4	7.78	0.0412	0.654
Inbred	C57	C57 4	PFA	1	237	4.62	7.73	0.0232	0.357
Inbred	CBA	CBA 1	PFA	1	177	3.69	8.72	0.0242	0.322
Inbred	CBA	CBA 2	PFA	1	308	3.89	8.71	0.0193	0.339
Inbred	CBA	CBA 3	MeAc	1	242	10.7	8.55	0.0587	0.912
Inbred	CBA	CBA 3	PFA	1	244	5.35	8.35	0.0286	0.447
Inbred	DBA	DBA 1	PFA	1	244	3.62	8.56	0.0198	0.31
Inbred	DBA	DBA 2	PFA	1	251	2.9	8.66	0.0158	0.251

Inbred	FVB	FVB 1	MeAc	1	216	14.5	7.96	0.0785	1.15
Inbred	FVB	FVB 1	PFA	1	298	3.86	7.75	0.0174	0.3
F1 hybrid	B6CBA	B6CBA 1	PFA	1	324	2.72	8.84	0.0133	0.24
F1 hybrid	B6CBA	B6CBA 2	PFA	1	313	3.08	8.64	0.015	0.266
F1 hybrid	B6CBA	B6CBA 4	PFA	1	218	3.29	8.55	0.019	0.281
F1 hybrid	CBAB6	CBAB6 1	PFA	1	217	2.8	8.53	0.0162	0.239
F1 hybrid	CBAB6	CBAB6 2	PFA	1	215	2.74	8.51	0.0159	0.233
F1 hybrid	CBAB6	CBAB6 3	PFA	1	232	2.16	8.75	0.0124	0.189
F1 hybrid	CBAB6	CBAB6 4	PFA	1	223	2.15	8.82	0.0127	0.189
Outbred	CD1	CD1 1	MeAc	15	260	11	8.73	0.0594	0.958
Outbred	CD1	CD1 1	PFA	15	229	4.03	8.68	0.0231	0.35
Outbred	MF1YRIII	MF1YRIII 2	MeAc	8	277	13.6	8.93	0.0729	1.21
Outbred	MF1YRIII	MF1YRIII 2	PFA	8	217	3.09	8.57	0.018	0.265
Wild-derived	LEWES	LEWES 1	PFA	2	273	3.11	8.58	0.0161	0.266
Wild-derived	LEWES	LEWES 2	PFA	2	278	3.17	8.53	0.0162	0.271
Wild-derived	PWK	PWK 2	PFA	2	275	2.8	8.81	0.0149	0.247
Wild-derived	PWK	PWK 3	PFA	2	268	3.85	8.78	0.0207	0.338
Wild-derived	STF	STF 1	PFA	2	288	2.75	7.6	0.0123	0.209
Wild-derived	STF	STF 2	PFA	2	295	2.46	7.58	0.0109	0.187

						**Min diameter across centre-of-mass (microns)**			
Status	Strain	Sample	Fixative	Number of animals pooled	Number of nuclei analysed	Coefficient of Variation	Mean	Standard error of the mean	Standard Deviation
Inbred	Balbc	Balbc 1	MeAc	1	201	14	3.35	0.0331	0.47
Inbred	Balbc	Balbc 1	PFA	1	281	9.72	3.31	0.0192	0.321
Inbred	Balbc	Balbc 2	MeAc	1	219	11	3.22	0.024	0.355
Inbred	Balbc	Balbc 2	PFA	1	293	9.85	3.28	0.0188	0.323
Inbred	C57	C57 3	MeAc	1	253	5.85	3.42	0.0126	0.2
Inbred	C57	C57 3	PFA	1	213	5.58	3.23	0.0124	0.18
Inbred	C57	C57 4	MeAc	1	252	6.67	3.36	0.0141	0.224
Inbred	C57	C57 4	PFA	1	237	5.26	3.19	0.0109	0.168
Inbred	CBA	CBA 1	PFA	1	177	3.88	3.5	0.0102	0.136
Inbred	CBA	CBA 2	PFA	1	308	4.86	3.46	0.00957	0.168
Inbred	CBA	CBA 3	MeAc	1	242	7.7	3.55	0.0176	0.273
Inbred	CBA	CBA 3	PFA	1	244	4.36	3.46	0.00965	0.151
Inbred	DBA	DBA 1	PFA	1	244	4.33	3.57	0.0099	0.155
Inbred	DBA	DBA 2	PFA	1	251	3.4	3.58	0.00768	0.122
Inbred	FVB	FVB 1	MeAc	1	216	8.82	3.06	0.0184	0.27
Inbred	FVB	FVB 1	PFA	1	298	4.76	3.19	0.00878	0.152
F1 hybrid	B6CBA	B6CBA 1	PFA	1	324	3.84	3.35	0.00714	0.129
F1 hybrid	B6CBA	B6CBA 2	PFA	1	313	4.26	3.38	0.00815	0.144
F1 hybrid	B6CBA	B6CBA 4	PFA	1	218	3.74	3.42	0.00867	0.128
F1 hybrid	CBAB6	CBAB6 1	PFA	1	217	3.51	3.45	0.00821	0.121
F1 hybrid	CBAB6	CBAB6 2	PFA	1	215	2.93	3.55	0.00708	0.104
F1 hybrid	CBAB6	CBAB6 3	PFA	1	232	2.94	3.58	0.00691	0.105
F1 hybrid	CBAB6	CBAB6 4	PFA	1	223	2.81	3.6	0.00677	0.101
Outbred	CD1	CD1 1	MeAc	15	260	8.27	3.11	0.016	0.257
Outbred	CD1	CD1 1	PFA	15	229	4.11	3.15	0.00856	0.13
Outbred	MF1YRIII	MF1YRIII 2	MeAc	8	277	11.7	3.39	0.0238	0.396
Outbred	MF1YRIII	MF1YRIII 2	PFA	8	217	5.18	3.41	0.012	0.176
Wild-derived	LEWES	LEWES 1	PFA	2	273	2.88	3.22	0.00561	0.0927
Wild-derived	LEWES	LEWES 2	PFA	2	278	2.92	3.21	0.00562	0.0938
Wild-derived	PWK	PWK 2	PFA	2	275	3.32	3.38	0.00676	0.112
Wild-derived	PWK	PWK 3	PFA	2	268	2.99	3.34	0.0061	0.0999
Wild-derived	STF	STF 1	PFA	2	288	3.67	3.51	0.00759	0.129
Wild-derived	STF	STF 2	PFA	2	295	2.94	3.49	0.00597	0.103

						Perimeter (microns)			
Status	Strain	Sample	Fixative	Number of animals pooled	Number of nuclei analysed	Coefficient of Variation	Mean	Standard error of the mean	Standard Deviation
Inbred	Balbc	Balbc 1	MeAc	1	201	17	20.6	0.248	3.51
Inbred	Balbc	Balbc 1	PFA	1	281	8.22	19.8	0.0972	1.63
Inbred	Balbc	Balbc 2	MeAc	1	219	17	20.3	0.233	3.45
Inbred	Balbc	Balbc 2	PFA	1	293	7.96	19.7	0.0914	1.57
Inbred	C57	C57 3	MeAc	1	253	5.7	20.3	0.0727	1.16
Inbred	C57	C57 3	PFA	1	213	4.27	20.4	0.0596	0.869
Inbred	C57	C57 4	MeAc	1	252	5.97	20.8	0.0781	1.24
Inbred	C57	C57 4	PFA	1	237	3.95	20.6	0.0528	0.812
Inbred	CBA	CBA 1	PFA	1	177	3.59	22.2	0.0599	0.796
Inbred	CBA	CBA 2	PFA	1	308	3.41	22.2	0.043	0.755
Inbred	CBA	CBA 3	MeAc	1	242	10.2	21.9	0.144	2.23
Inbred	CBA	CBA 3	PFA	1	244	4.64	21.3	0.0633	0.989
Inbred	DBA	DBA 1	PFA	1	244	2.59	22.4	0.0372	0.581
Inbred	DBA	DBA 2	PFA	1	251	2.49	22.7	0.0356	0.564
Inbred	FVB	FVB 1	MeAc	1	216	17.9	20.7	0.252	3.7
Inbred	FVB	FVB 1	PFA	1	298	3.47	20	0.0403	0.695
F1 hybrid	B6CBA	B6CBA 1	PFA	1	324	2.71	22.7	0.0341	0.615
F1 hybrid	B6CBA	B6CBA 2	PFA	1	313	2.94	22.2	0.0369	0.653
F1 hybrid	B6CBA	B6CBA 4	PFA	1	218	3	22.1	0.0448	0.662
F1 hybrid	CBAB6	CBAB6 1	PFA	1	217	2.53	21.9	0.0377	0.555
F1 hybrid	CBAB6	CBAB6 2	PFA	1	215	2.39	21.8	0.0355	0.521
F1 hybrid	CBAB6	CBAB6 3	PFA	1	232	2.04	22.4	0.03	0.457
F1 hybrid	CBAB6	CBAB6 4	PFA	1	223	1.92	22.6	0.0291	0.434
Outbred	CD1	CD1 1	MeAc	15	260	13	21.8	0.176	2.84
Outbred	CD1	CD1 1	PFA	15	229	3.62	22	0.0527	0.797
Outbred	MF1YRIII	MF1YRIII 2	MeAc	8	277	20.1	23.7	0.286	4.76
Outbred	MF1YRIII	MF1YRIII 2	PFA	8	217	3	22.4	0.0457	0.674
Wild-derived	LEWES	LEWES 1	PFA	2	273	2.98	21.5	0.0388	0.641
Wild-derived	LEWES	LEWES 2	PFA	2	278	2.82	21.4	0.0363	0.605
Wild-derived	PWK	PWK 2	PFA	2	275	2.68	21.8	0.0352	0.584
Wild-derived	PWK	PWK 3	PFA	2	268	3.61	21.7	0.0478	0.783
Wild-derived	STF	STF 1	PFA	2	288	2.88	20.2	0.0342	0.581
Wild-derived	STF	STF 2	PFA	2	295	2.46	20.1	0.0289	0.496

						Regularity			
Status	Strain	Sample	Fixative	Number of animals pooled	Number of nuclei analysed	Coefficient of Variation	Mean	Standard error of the mean	Standard Deviation
Inbred	Balbc	Balbc 1	MeAc	1	201	11.9	1.55	0.013	0.184
Inbred	Balbc	Balbc 1	PFA	1	281	9.72	1.55	0.00897	0.15
Inbred	Balbc	Balbc 2	MeAc	1	219	11.1	1.57	0.0117	0.174
Inbred	Balbc	Balbc 2	PFA	1	293	9.88	1.53	0.00884	0.151
Inbred	C57	C57 3	MeAc	1	253	6.9	1.48	0.00644	0.102
Inbred	C57	C57 3	PFA	1	213	6.03	1.57	0.00649	0.0948
Inbred	C57	C57 4	MeAc	1	252	8	1.53	0.0077	0.122
Inbred	C57	C57 4	PFA	1	237	6.19	1.57	0.00632	0.0973
Inbred	CBA	CBA 1	PFA	1	177	5.28	1.5	0.00597	0.0794
Inbred	CBA	CBA 2	PFA	1	308	7.35	1.56	0.00655	0.115
Inbred	CBA	CBA 3	MeAc	1	242	8.95	1.43	0.0082	0.128
Inbred	CBA	CBA 3	PFA	1	244	7.57	1.44	0.00699	0.109
Inbred	DBA	DBA 1	PFA	1	244	5.25	1.61	0.00542	0.0846
Inbred	DBA	DBA 2	PFA	1	251	4.49	1.62	0.00458	0.0726

Inbred	FVB	FVB 1	MeAc	1	216	14.2	1.54	0.0148	0.218
Inbred	FVB	FVB 1	PFA	1	298	7.65	1.55	0.00687	0.119
F1 hybrid	B6CBA	B6CBA 1	PFA	1	324	4.2	1.6	0.00373	0.0672
F1 hybrid	B6CBA	B6CBA 2	PFA	1	313	5.22	1.56	0.00461	0.0815
F1 hybrid	B6CBA	B6CBA 4	PFA	1	218	5.95	1.52	0.00613	0.0905
F1 hybrid	CBAB6	CBAB6 1	PFA	1	217	5.36	1.55	0.00565	0.0832
F1 hybrid	CBAB6	CBAB6 2	PFA	1	215	5.2	1.46	0.00518	0.0759
F1 hybrid	CBAB6	CBAB6 3	PFA	1	232	3.59	1.5	0.00354	0.054
F1 hybrid	CBAB6	CBAB6 4	PFA	1	223	4.18	1.51	0.00422	0.063
Outbred	CD1	CD1 1	MeAc	15	260	11.2	1.65	0.0115	0.185
Outbred	CD1	CD1 1	PFA	15	229	7.07	1.66	0.00776	0.117
Outbred	MF1YRIII	MF1YRIII 2	MeAc	8	277	11	1.69	0.0112	0.186
Outbred	MF1YRIII	MF1YRIII 2	PFA	8	217	5.5	1.65	0.00617	0.0909
Wild-derived	LEWES	LEWES 1	PFA	2	273	5.49	1.65	0.00547	0.0904
Wild-derived	LEWES	LEWES 2	PFA	2	278	5.14	1.65	0.00509	0.0849
Wild-derived	PWK	PWK 2	PFA	2	275	4.68	1.4	0.00396	0.0657
Wild-derived	PWK	PWK 3	PFA	2	268	4.79	1.47	0.00431	0.0705
Wild-derived	STF	STF 1	PFA	2	288	4.37	1.46	0.00377	0.0639
Wild-derived	STF	STF 2	PFA	2	295	4.08	1.47	0.00349	0.06

						**Width of body (microns)**			
Status	Strain	Sample	Fixative	Number of animals pooled	Number of nuclei analysed	Coefficient of Variation	Mean	Standard error of the mean	Standard Deviation
Inbred	Balbc	Balbc 1	MeAc	1	201	14.1	3.72	0.037	0.524
Inbred	Balbc	Balbc 1	PFA	1	281	18.2	3.59	0.039	0.654
Inbred	Balbc	Balbc 2	MeAc	1	219	20.8	3.6	0.0505	0.748
Inbred	Balbc	Balbc 2	PFA	1	293	16.5	3.53	0.0341	0.583
Inbred	C57	C57 3	MeAc	1	253	8.25	3.65	0.0189	0.301
Inbred	C57	C57 3	PFA	1	213	6.43	3.4	0.015	0.218
Inbred	C57	C57 4	MeAc	1	252	11.1	3.6	0.0251	0.398
Inbred	C57	C57 4	PFA	1	237	5.78	3.33	0.0125	0.192
Inbred	CBA	CBA 1	PFA	1	177	6.22	4.71	0.022	0.293
Inbred	CBA	CBA 2	PFA	1	308	5.87	3.56	0.0119	0.209
Inbred	CBA	CBA 3	MeAc	1	242	11.5	4.49	0.0333	0.518
Inbred	CBA	CBA 3	PFA	1	244	7.65	4.47	0.0219	0.342
Inbred	DBA	DBA 1	PFA	1	244	5.5	3.72	0.0131	0.205
Inbred	DBA	DBA 2	PFA	1	251	4.4	3.74	0.0104	0.165
Inbred	FVB	FVB 1	MeAc	1	216	21.4	4.18	0.0607	0.892
Inbred	FVB	FVB 1	PFA	1	298	9.75	3.36	0.019	0.328
F1 hybrid	B6CBA	B6CBA 1	PFA	1	324	4.63	4.91	0.0126	0.227
F1 hybrid	B6CBA	B6CBA 2	PFA	1	313	5.47	3.47	0.0107	0.19
F1 hybrid	B6CBA	B6CBA 4	PFA	1	218	5.69	4.82	0.0186	0.274
F1 hybrid	CBAB6	CBAB6 1	PFA	1	217	4.68	3.55	0.0113	0.166
F1 hybrid	CBAB6	CBAB6 2	PFA	1	215	3.27	3.66	0.00815	0.119
F1 hybrid	CBAB6	CBAB6 3	PFA	1	232	3.81	3.69	0.00922	0.14
F1 hybrid	CBAB6	CBAB6 4	PFA	1	223	3.08	3.71	0.00764	0.114
Outbred	CD1	CD1 1	MeAc	15	260	15.5	3.23	0.0311	0.501
Outbred	CD1	CD1 1	PFA	15	229	4.94	3.25	0.0106	0.16
Outbred	MF1YRIII	MF1YRIII 2	MeAc	8	277	21.8	3.65	0.0478	0.796
Outbred	MF1YRIII	MF1YRIII 2	PFA	8	217	5.3	3.57	0.0128	0.189
Wild-derived	LEWES	LEWES 1	PFA	2	273	3.65	3.31	0.00731	0.121
Wild-derived	LEWES	LEWES 2	PFA	2	278	4.09	3.32	0.00813	0.135
Wild-derived	PWK	PWK 2	PFA	2	275	6.95	3.55	0.0149	0.247
Wild-derived	PWK	PWK 3	PFA	2	268	6.06	4.21	0.0156	0.255
Wild-derived	STF	STF 1	PFA	2	288	4.62	3.74	0.0102	0.173
Wild-derived	STF	STF 2	PFA	2	295	3.85	3.73	0.00836	0.144
