# Supplementary material for: A high-throughput method for unbiased quantitation and categorization of nuclear morphology
Source: Biol Reprod. 2019 Feb 11;100(5):1250–60. doi: 10.1093/biolre/ioz013 (PMC6497523; doi:10.1093/biolre/ioz013)
Supplement: ioz013_Supplemental_Files [file ioz013_supplemental_files.zip › Supplementary table 3.pdf]

Sample	Segment	Number of nuclei	Mean length (microns)	Length standard deviation	Length coefficient of variation	Length standard error of the mean
B6CBA 1	Segment 0	324	1.75	0.116	6.66	0.00647
B6CBA 1	Segment 1	324	5.46	0.288	5.26	0.016
B6CBA 1	Segment 2	324	1.36	0.159	11.7	0.00881
B6CBA 1	Segment 3	324	1.67	0.206	12.4	0.0114
B6CBA 1	Segment 4	324	12.8	0.379	2.96	0.0211
B6CBA 2	Segment 0	313	1.69	0.117	6.92	0.0066
B6CBA 2	Segment 1	313	5.37	0.269	5.02	0.0152
B6CBA 2	Segment 2	313	1.31	0.116	8.87	0.00656
B6CBA 2	Segment 3	313	1.56	0.235	15	0.0133
B6CBA 2	Segment 4	313	12.6	0.407	3.22	0.023
B6CBA 4	Segment 0	218	1.75	0.123	7	0.00831
B6CBA 4	Segment 1	218	5.22	0.309	5.92	0.0209
B6CBA 4	Segment 2	218	1.35	0.122	9.05	0.00826
B6CBA 4	Segment 3	218	1.54	0.196	12.8	0.0133
B6CBA 4	Segment 4	218	12.5	0.379	3.02	0.0257
BALB/c 1	Segment 0	281	2.7	0.363	13.5	0.0217
BALB/c 1	Segment 1	281	3.88	0.756	19.5	0.0451
BALB/c 1	Segment 2	281	2.12	0.692	32.6	0.0413
BALB/c 1	Segment 3	281	2.55	0.602	23.6	0.0359
BALB/c 1	Segment 4	281	8.89	1.23	13.8	0.0732
BALB/c 2	Segment 0	293	2.8	0.41	14.6	0.0239
BALB/c 2	Segment 1	293	3.77	0.703	18.6	0.041
BALB/c 2	Segment 2	293	2.19	0.704	32.1	0.0411
BALB/c 2	Segment 3	293	2.41	0.618	25.6	0.0361
BALB/c 2	Segment 4	293	8.83	1.25	14.2	0.0733
C57 3	Segment 0	213	2.66	0.22	8.24	0.015
C57 3	Segment 1	213	3.94	0.407	10.3	0.0279
C57 3	Segment 2	213	1.15	0.143	12.4	0.00983
C57 3	Segment 3	213	1.23	0.172	14	0.0118
C57 3	Segment 4	213	2.29	0.326	14.3	0.0224
C57 3	Segment 5	213	9.49	0.606	6.39	0.0415
C57 4	Segment 0	237	2.69	0.215	7.98	0.014
C57 4	Segment 1	237	4.05	0.378	9.33	0.0246
C57 4	Segment 2	237	1.14	0.0951	8.31	0.00617
C57 4	Segment 3	237	1.21	0.124	10.3	0.00806
C57 4	Segment 4	237	2.29	0.387	16.9	0.0252
C57 4	Segment 5	237	9.57	0.639	6.68	0.0415
CBA 2	Segment 0	308	1.87	0.13	6.96	0.0074
CBA 2	Segment 1	308	2.59	0.154	5.94	0.00875
CBA 2	Segment 2	308	2.65	0.176	6.65	0.01
CBA 2	Segment 3	308	1.36	0.148	10.9	0.00846
CBA 2	Segment 4	308	1.77	0.276	15.5	0.0157
CBA 2	Segment 5	308	12.3	0.478	3.87	0.0272
CBA 3	Segment 0	244	1.9	0.132	6.98	0.00847
CBA 3	Segment 1	244	2.48	0.217	8.77	0.0139
CBA 3	Segment 2	244	2.49	0.241	9.69	0.0154
CBA 3	Segment 3	244	1.33	0.208	15.6	0.0133
CBA 3	Segment 4	244	1.6	0.327	20.4	0.0209
CBA 3	Segment 5	244	11.9	0.553	4.63	0.0354
CBAB6 1	Segment 0	217	1.75	0.0925	5.3	0.00628
CBAB6 1	Segment 1	217	5.19	0.22	4.25	0.015
CBAB6 1	Segment 2	217	1.34	0.114	8.53	0.00774

CBAB6 1	Segment 3	217	1.55	0.173	11.1	0.0117
CBAB6 1	Segment 4	217	12.4	0.333	2.67	0.0226
CBAB6 2	Segment 0	215	1.76	0.0921	5.23	0.00628
CBAB6 2	Segment 1	215	5.01	0.248	4.94	0.0169
CBAB6 2	Segment 2	215	1.31	0.128	9.78	0.00872
CBAB6 2	Segment 3	215	1.53	0.174	11.4	0.0119
CBAB6 2	Segment 4	215	12.5	0.301	2.4	0.0205
CBAB6 3	Segment 0	232	1.77	0.096	5.41	0.0063
CBAB6 3	Segment 1	232	5.18	0.232	4.48	0.0152
CBAB6 3	Segment 2	232	1.35	0.156	11.5	0.0102
CBAB6 3	Segment 3	232	1.67	0.167	9.96	0.0109
CBAB6 3	Segment 4	232	12.8	0.277	2.17	0.0182
CBAB6 4	Segment 0	223	1.85	0.0978	5.3	0.00655
CBAB6 4	Segment 1	223	5.13	0.228	4.45	0.0153
CBAB6 4	Segment 2	223	1.39	0.112	8.04	0.00747
CBAB6 4	Segment 3	223	1.7	0.166	9.77	0.0111
CBAB6 4	Segment 4	223	12.8	0.271	2.11	0.0182
CD1 1	Segment 0	229	1.76	0.117	6.65	0.00772
CD1 1	Segment 1	229	5.27	0.411	7.79	0.0271
CD1 1	Segment 2	229	1.27	0.136	10.7	0.009
CD1 1	Segment 3	229	1.63	0.273	16.7	0.018
CD1 1	Segment 4	229	12.4	0.486	3.91	0.0321
DBA 1	Segment 0	244	1.81	0.11	6.04	0.00701
DBA 1	Segment 1	244	5.32	0.335	6.29	0.0214
DBA 1	Segment 2	244	1.34	0.186	13.9	0.0119
DBA 1	Segment 3	244	1.48	0.274	18.6	0.0175
DBA 1	Segment 4	244	4.14	0.572	13.8	0.0366
DBA 1	Segment 5	244	8.77	0.581	6.63	0.0372
DBA 2	Segment 0	251	1.79	0.0946	5.29	0.00597
DBA 2	Segment 1	251	5.28	0.276	5.22	0.0174
DBA 2	Segment 2	251	1.36	0.142	10.5	0.00895
DBA 2	Segment 3	251	1.57	0.208	13.3	0.0131
DBA 2	Segment 4	251	4.29	0.446	10.4	0.0281
DBA 2	Segment 5	251	8.81	0.438	4.97	0.0276
FVB 1	Segment 0	298	1.73	0.114	6.59	0.00659
FVB 1	Segment 1	298	2.5	0.143	5.7	0.00827
FVB 1	Segment 2	298	2.58	0.166	6.45	0.00964
FVB 1	Segment 3	298	2.29	0.183	8	0.0106
FVB 1	Segment 4	298	2.03	0.297	14.6	0.0172
FVB 1	Segment 5	298	9.29	0.497	5.36	0.0288
LEWES 1	Segment 0	273	2.1	0.121	5.76	0.00732
LEWES 1	Segment 1	273	2.41	0.151	6.27	0.00913
LEWES 1	Segment 2	273	2.4	0.173	7.2	0.0105
LEWES 1	Segment 3	273	1.32	0.13	9.84	0.00787
LEWES 1	Segment 4	273	1.74	0.249	14.3	0.015
LEWES 1	Segment 5	273	12	0.406	3.4	0.0246
LEWES 2	Segment 0	278	2.14	0.113	5.27	0.00675
LEWES 2	Segment 1	278	2.39	0.107	4.47	0.0064
LEWES 2	Segment 2	278	2.38	0.134	5.64	0.00805
LEWES 2	Segment 3	278	1.33	0.114	8.59	0.00685
LEWES 2	Segment 4	278	1.71	0.202	11.9	0.0121
LEWES 2	Segment 5	278	11.9	0.319	2.68	0.0192
MF1YRIII 2	Segment 0	217	2.51	0.19	7.57	0.0129
MF1YRIII 2	Segment 1	217	4.78	0.342	7.15	0.0232
MF1YRIII 2	Segment 2	217	1.23	0.155	12.5	0.0105
MF1YRIII 2	Segment 3	217	1.33	0.241	18	0.0163

MF1YRIII 2	Segment 4	217	1.99	0.396	20	0.0269
MF1YRIII 2	Segment 5	217	11	0.554	5.04	0.0376
PWK 2	Segment 0	275	1.92	0.119	6.2	0.00716
PWK 2	Segment 1	275	2.52	0.144	5.72	0.00869
PWK 2	Segment 2	275	2.59	0.166	6.42	0.01
PWK 2	Segment 3	275	1.36	0.195	14.3	0.0118
PWK 2	Segment 4	275	1.72	0.24	13.9	0.0145
PWK 2	Segment 5	275	12.1	0.402	3.32	0.0243
PWK 3	Segment 0	268	1.82	0.128	7.04	0.00784
PWK 3	Segment 1	268	2.55	0.177	6.96	0.0108
PWK 3	Segment 2	268	2.61	0.199	7.6	0.0121
PWK 3	Segment 3	268	1.39	0.181	13	0.0111
PWK 3	Segment 4	268	1.68	0.256	15.3	0.0157
PWK 3	Segment 5	268	12	0.581	4.82	0.0355
STF 1	Segment 0	288	1.76	0.105	5.99	0.0062
STF 1	Segment 1	288	2.11	0.412	19.6	0.0243
STF 1	Segment 2	288	2.02	0.367	18.1	0.0216
STF 1	Segment 3	288	1.32	0.178	13.4	0.0105
STF 1	Segment 4	288	1.5	0.226	15.1	0.0133
STF 1	Segment 5	288	1.76	0.493	28	0.0291
STF 1	Segment 6	288	10.2	0.486	4.78	0.0286
STF 2	Segment 0	295	1.82	0.123	6.76	0.00716
STF 2	Segment 1	295	2.02	0.109	5.37	0.00632
STF 2	Segment 2	295	2.02	0.132	6.56	0.00771
STF 2	Segment 3	295	1.27	0.107	8.41	0.0062
STF 2	Segment 4	295	1.53	0.163	10.6	0.00947
STF 2	Segment 5	295	1.83	0.4	21.8	0.0233
STF 2	Segment 6	295	10.1	0.458	4.53	0.0267
