# Supplementary material for: A high-throughput method for unbiased quantitation and categorization of nuclear morphology
Source: Biol Reprod. 2019 Feb 11;100(5):1250–60. doi: 10.1093/biolre/ioz013 (PMC6497523; doi:10.1093/biolre/ioz013)
Supplement: ioz013_Supplemental_Files [file ioz013_supplemental_files.zip › Supplementary_methods_results.pdf]

Supplementary data

This is a supplementary data file for Skinner et al: A high-throughput method for unbiased quantitation and categorisation of nuclear morphology.

Large supplementary tables are presented in separate data files:

Supplementary Table 1: Measured nuclear parameters by strain
Supplementary Table 2: Measured nuclear parameters by sample
Supplementary Table 3: The default mean segment lengths by strain, with coefficient of variability, standard error and standard deviation per segment

Table of Contents

Supplementary Methods **2**
- Detection of nuclei 3
  - Detecting nuclei - the chromocenter 3
- Morphological analysis of nuclei 4
  - Generation of angle profiles 4
  - The perimeter proportion used for generating shape profiles 5
  - Landmark detection 5
  - Segmentation of profiles 6
- Other scripts and resources 8

Supplementary Results **9**
- Nuclear size and shape is largely independent of fixation method 9
- Shape analysis is not specific to the microscope or camera used for imaging 11
- Automatic camera exposure times are sufficient for robust nucleus detection 13
- Individual samples are consistent with strain averages 17
- Patterns of default profile segmentation varying by strain 18
- Manual versus automated clustering 20
- Comparison of our measurements with previous studies 22
- Clustered BALB/c nuclei match published phenotypic descriptions 24

Supplementary References **25**

Supplementary Methods

The core software was developed in Java 8, with the user interface written using Swing for cross-platform compatibility. The analysis flow is given in Supplementary Figure 1 below; we have designed the analysis to run almost entirely automatedly after images have been captured, with only an initial setup of nucleus detection parameters required.

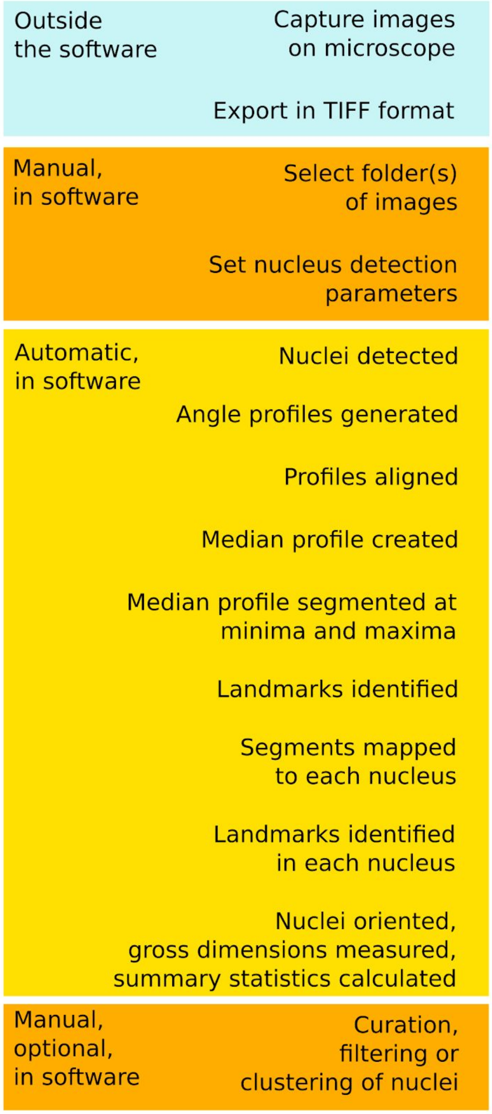

Supplementary figure 1: The analysis pipeline. The majority of the analysis is entirely automatic after detection parameters have been set. Each step is outlined further in this document. Optionally, the detected nuclei can be manually curated to remove poorly detected cells.

Detection of nuclei

Images were exported from SmartCapture in TIFF format (Supplementary Figure 2A). A Kuwahara filter [1] was applied to remove background noise while preserving edges (Supplementary Figure 1B). Bright internal structures within nuclei (such as the chromocenter) were removed by thresholding the DAPI signal (Supplementary Figure 2C; Supplementary Figure 3). Nuclear edges were then detected using the Canny edge detection algorithm [2], a more robust means for finding edges than conventional threshold-based edge finding (Supplementary Figure 2D). A morphological closing operation was performed to fill breaks in the nuclear perimeters left by the edge detection (Supplementary Figure 2E), using the MorphoLibJ library [3], and the outlines of resulting complete objects were detected with the default ImageJ particle analyser (Supplementary Figure 2F). Basic filters were applied to ensure that only objects within bounds of area and circularity were considered as nuclei; these values are set during the analysis setup.

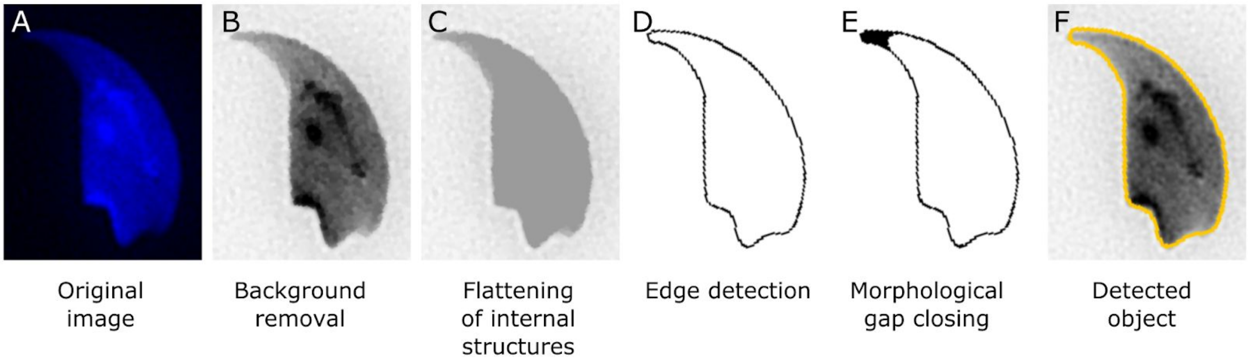

Supplementary Figure 2: The process of detecting nuclei: A) Input; B) Background removal; C) Chromocenter flattening; D) Edge detection; E) Gap closing; F) detected object. Images B-F have been inverted for clarity.

Detecting nuclei - the chromocenter

An edge detection issue we encountered with mouse sperm lies in the distinctive chromocenter. This region stains more intensely with DAPI than the surrounding chromatin, and so can cause the edge detector to ‘skip’, as shown in Supplementary Figure 3. To avoid this, we used a threshold-based flattening of the images, ensuring bright internal structures are not visible to the edge detector. A user-adjustable threshold was set (default 100), and any pixels in the image brighter than this were set to the threshold value.

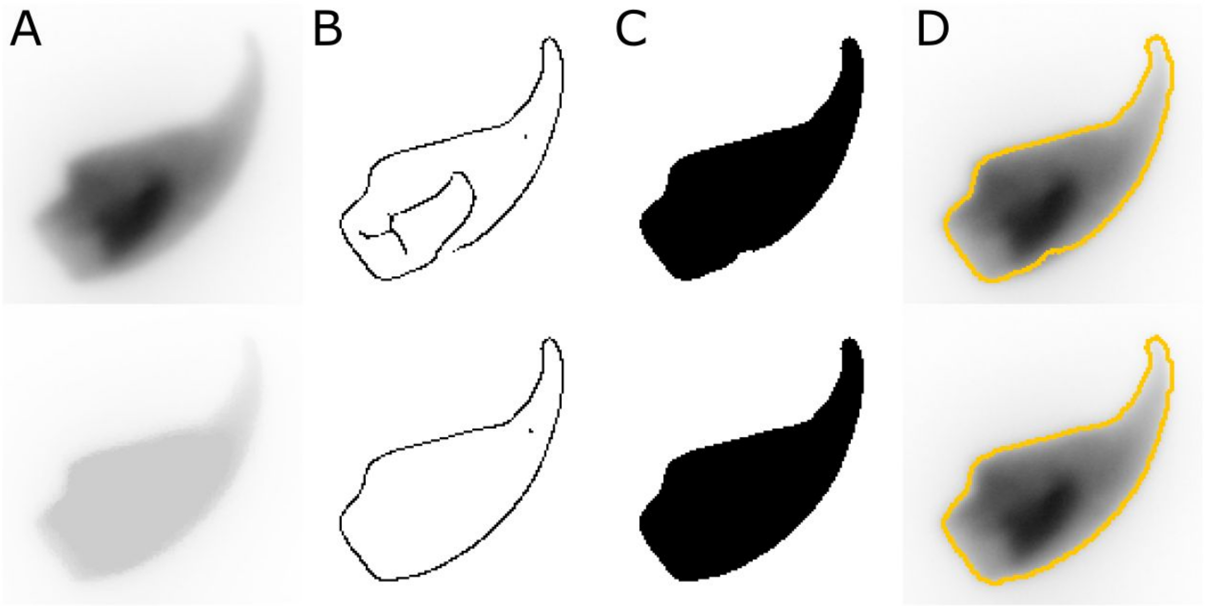

Supplementary Figure 2: Effect of removing chromocenters before edge detection. Upper panel: without chromocentre removal; lower panel; with chromocentre removal. The images show (A) the input to the edge detector, (B) the detected edges, (C) the gap closed image, and (D) the detected nucleus outline overlaid on the original image. Images have been inverted for clarity.

Morphological analysis of nuclei

Generation of angle profiles

The conventional Zahn-Roskies (Z-R) transform approximates a given shape as a polygon based on a fixed number of semilandmarks spaced evenly around the perimeter of the shape, and then measures the angle at each vertex of the resulting polygon [4].

In our analyses, we use the interior angle (as shown in Figure 1), and measure this across a window of 5% of the total object perimeter - equivalent to a Z-R transform with 20 semilandmarks per object. However, in contrast to the Z-R transform that uses a single set of semilandmarks per object and only measures the angle at each semilandmark, we instead measure the interior angle at points evenly spaced a single pixel apart around the shape's perimeter.

The final result is thus equivalent to combining multiple overlapping Z-R transforms, each offset by a single point so as not to duplicate or lose information. We find this to give a higher-resolution encoding of the shape that loses less information in finely-detailed areas such as the hook tip and tail attachment site.

The perimeter proportion used for generating shape profiles

The window size chosen for the angle profile analysis is important since it determines the size of morphological feature that can be resolved. Too small, and no features are visible. Too high, and subtle shapes will be lost. The software includes the ability to change the window size as needed, and to visualise the effects of using different window sizes on a population of cells. Our window size of 5% was chosen to be maximally informative, providing the best discrimination of features in the mouse sperm following testing of a range of values (Supplementary Figure 4).

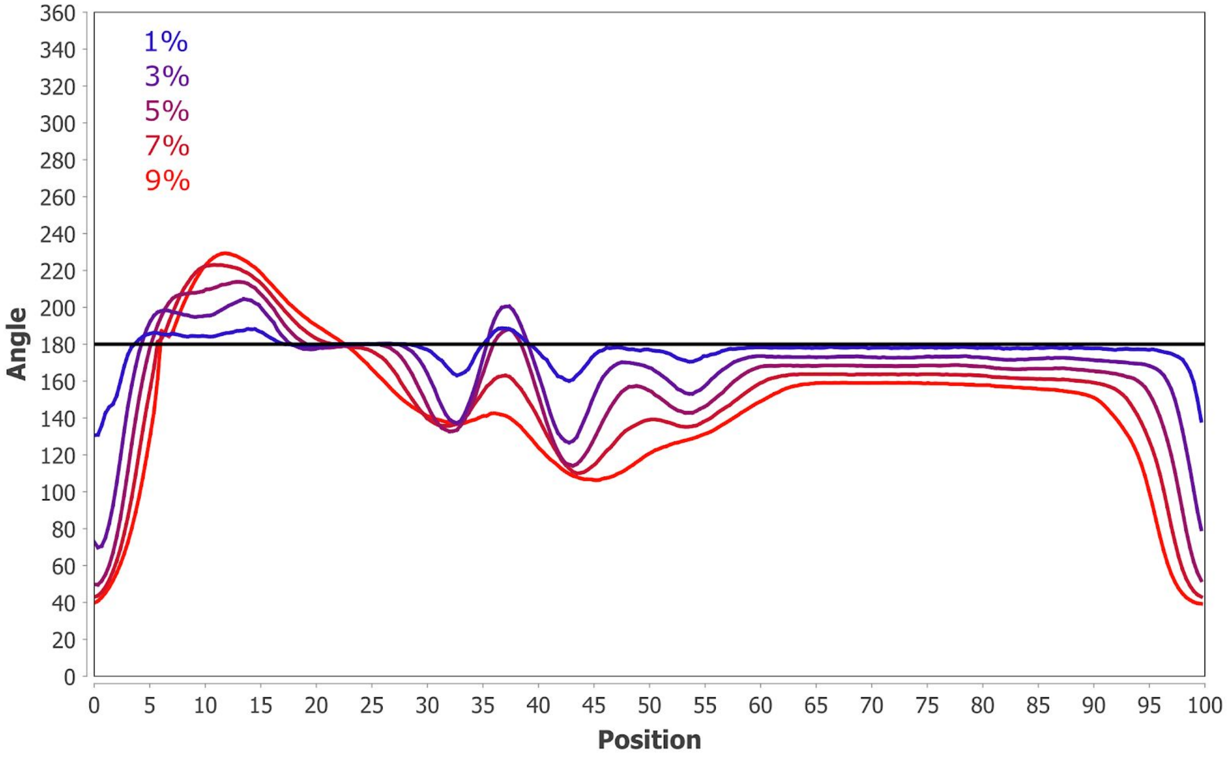

Supplementary Figure 4: Effect of varying window proportion for angle profile generation from 1% to 9% of the nuclear perimeter in a population of C57Bl6 sperm nuclei. By default, we use 5% of the perimeter, which allows optimal detection of the curvature of mouse sperm nuclei. At higher and lower proportions, detail of corners is lost.

Landmark detection

Following the generation of angle profiles from each 2D sperm image, the profiles from each nucleus can be aligned against each other, and features of interest detected. Since sperm may settle on the slide in either of two orientations (with the apical hook pointing to the left or right respectively), each profile is aligned against the median in both forward and reverse orientation - note that reversal of the angle profile is equivalent to reflection of the sperm image. This allows automatic rotation/reflection and consistent orientation of each nucleus for standardised analysis. In keeping with convention, we display nuclei with the apical hook pointing to the left,

and the flat portion of the sperm ventral surface oriented vertically. Note that since mouse sperm are almost entirely flat, they do not fall in other confounding orientations on the slide.

The nuclear profiles are normalised to an equal length and aligned against each other using the minimum sum-of-squares distance. A median profile is constructed and taken as representative of the population. The median profile is constructed by taking the median of the values in the aligned angle profiles at each point (Supplementary Figure 5).

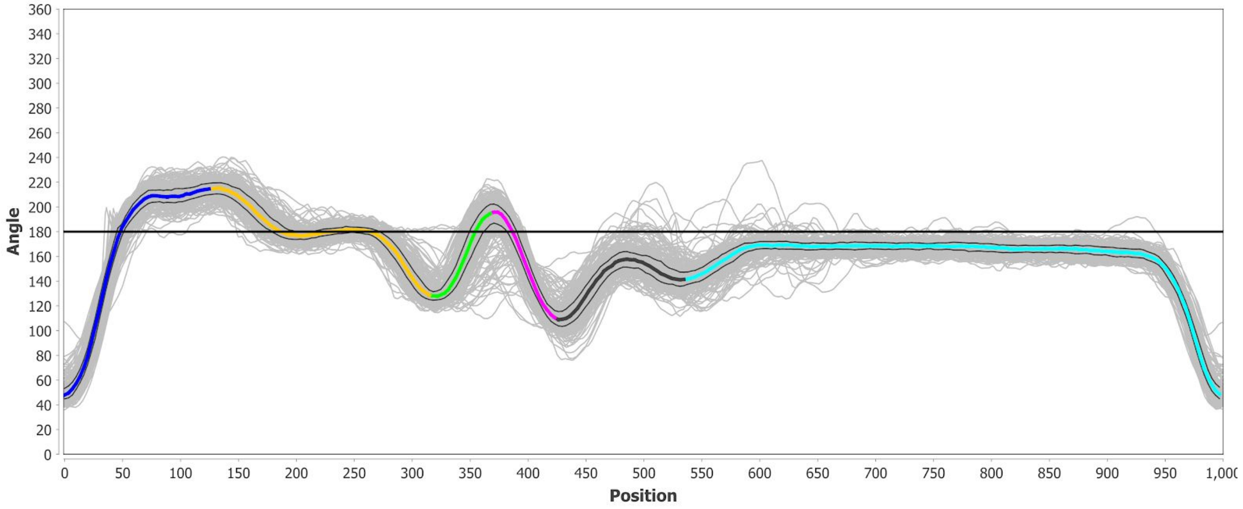

Supplementary Figure 5: Individual nucleus profiles (grey) and a segmented median. Profiles have been normalised to a consistent length.

The median angle profile allows automatic detection of Type II landmarks, defined as regions of maximum and minimum curvature within the outline [5]. These in turn correspond to regions of interest in relation to the morphodynamic processes shaping the sperm head. The median profile is segmented at local minima below 180 degrees and maxima above 180 degrees to generate the landmarks for feature recognition. Segment boundaries are propagated onto the profiles of the individual nuclei. A best fit alignment is calculated for each segment individually, to ensure the most appropriate segment endpoint is chosen in each nucleus and to account for potential differing segment sizes in different nuclei. This allows the features detected in the median profile to be located in each sperm nucleus, even when the shape outline is not optimal

Segmentation of profiles

The alignment of each nucleus profile to the median allows landmarks to still be found in nuclei with poor edge detection or abnormalities - for example in the outlier profiles in Supplementary Figure 5 above seen especially between positions 500 and 600.

Assigning segments to individual nuclei (i.e. mapping the position of landmarks identified in the

median profile back to each nucleus) requires remapping the segment end points as defined in the median to the best fitting region of the nucleus profile. The problem is illustrated in Supplementary Figure 6; if segments are propagated by proportional size in the profile, they may not correspond to actual maxima or minima in a nucleus - for example, the yellow segment below is smaller in the profile being segmented, and this would throw off all subsequent segment endpoints.

By running a best-fit alignment between the template yellow segment, and a range of possible target segments with greater or smaller endpoints, the optimal position can be chosen to map the segment boundaries. This approach also ensures that we do not rely on an ability to cleanly identify distinct maxima and minima in every nucleus profile; landmarks are identified at the population level.

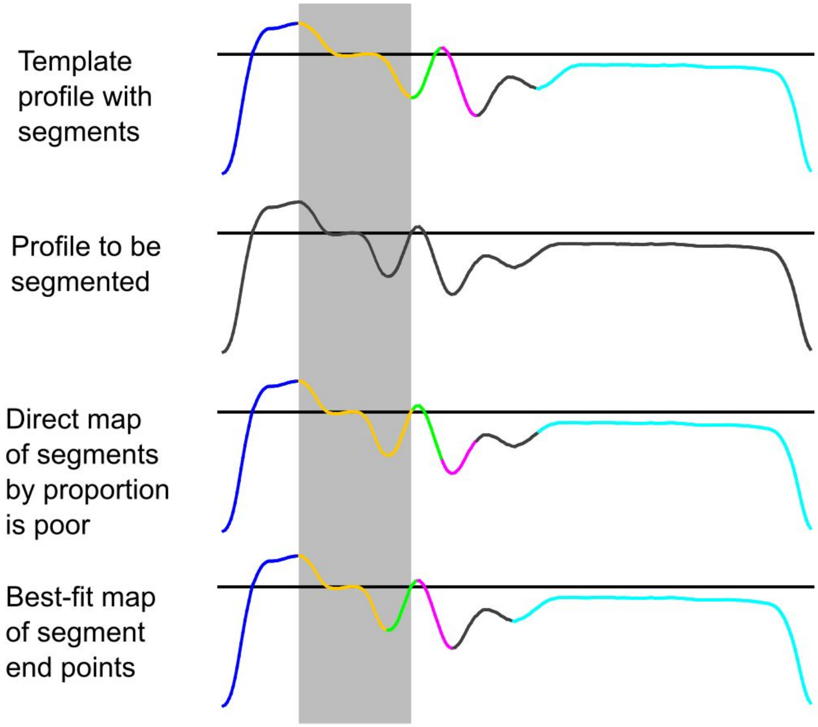

Supplementary Figure 6: Adjusting segment boundaries from the median to best fit individual nuclei. The segments from the template profile have been adjusted to fit the grey unsegmented profile, with the proportional bounds of the yellow segment in the template profile highlighted.

Other scripts and resources

Violin plots for figures were generated in R using ggplot, and finished in Inkscape 0.92 (https://inkscape.org). Clustering distance trees were visualised using FigTree 1.4.2 (http://tree.bio.ed.ac.uk/software/figtree/). An example script to generate the violin plots can be found at https://github.com/bmskinner/general/blob/master/plot_multiple_ggplots_demo.r, which will run on example data at https://github.com/bmskinner/general/blob/master/plot_multiple_ggplots_demo.csv

Example images of MF1Y^{RIII} nuclei for testing the software can be downloaded from https://bitbucket.org/bmskinner/nuclear_morphology/downloads/Testing_mouse_image_set.zip

Supplementary Results

Nuclear size and shape is largely independent of fixation method

We split samples from individual mice, and fixed half with methanol-acetic acid and the rest with 2% PFA. We then imaged the samples on the same microscopy system. The morphology analysis showed no differences in the angle profiles generated (Supplementary Figure 7), but MeAc-fixed samples were slightly different in two dimensional area than PFA-fixed samples, with the direction of the difference varying between strains (Supplementary Figure 8). This could be due to PFA preserving 3D structure more robustly, to the additional dehydration from the alcohol, or to other factors. Given the negligible differences in angle profile data between the two fixatives, we arbitrarily standardised on PFA-fixed sperm for the remainder of our analyses.

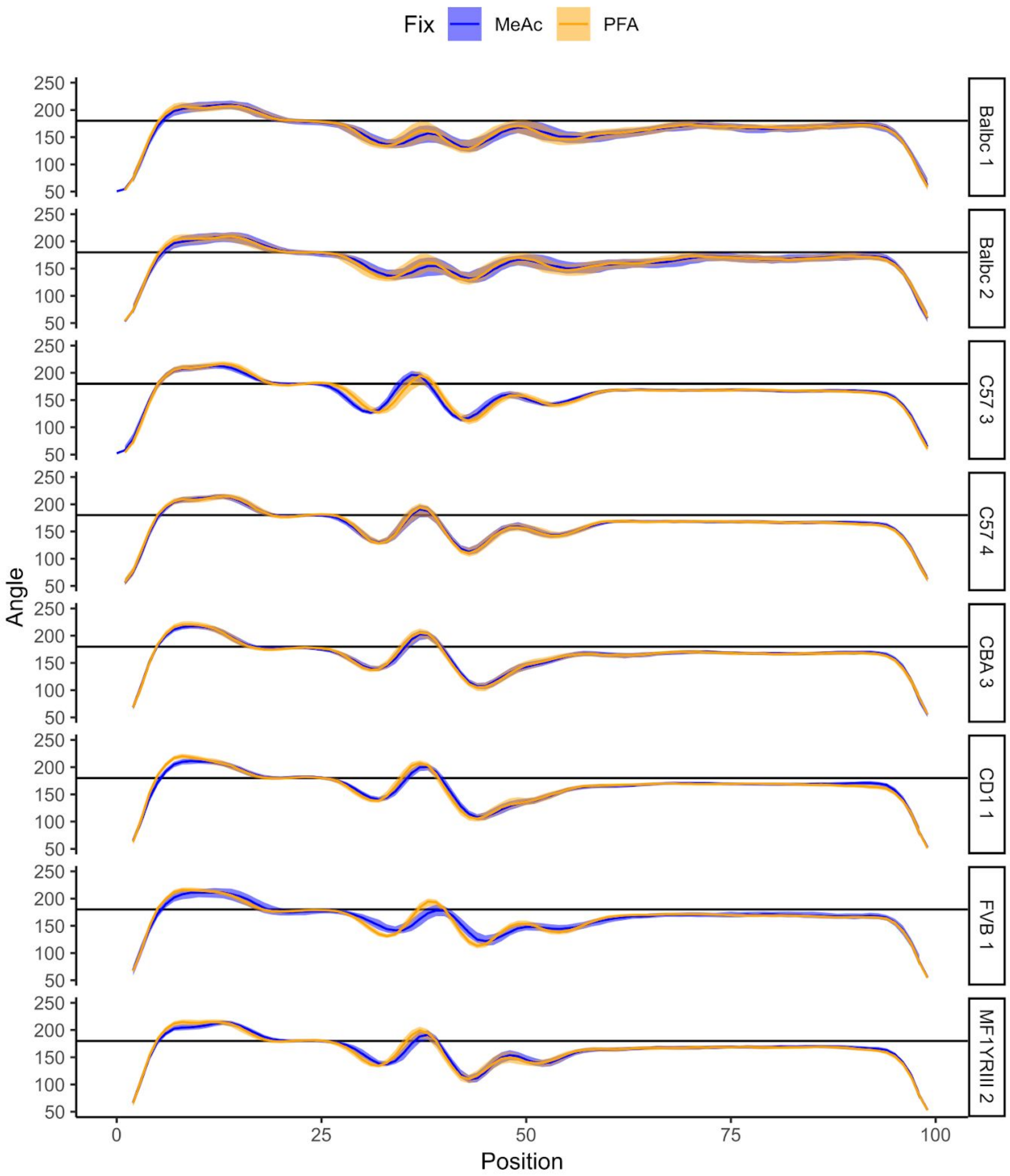

Supplementary Figure 7: Comparison of profiles from Me:Ac and PFA fixed sperm shows no difference in overall shape

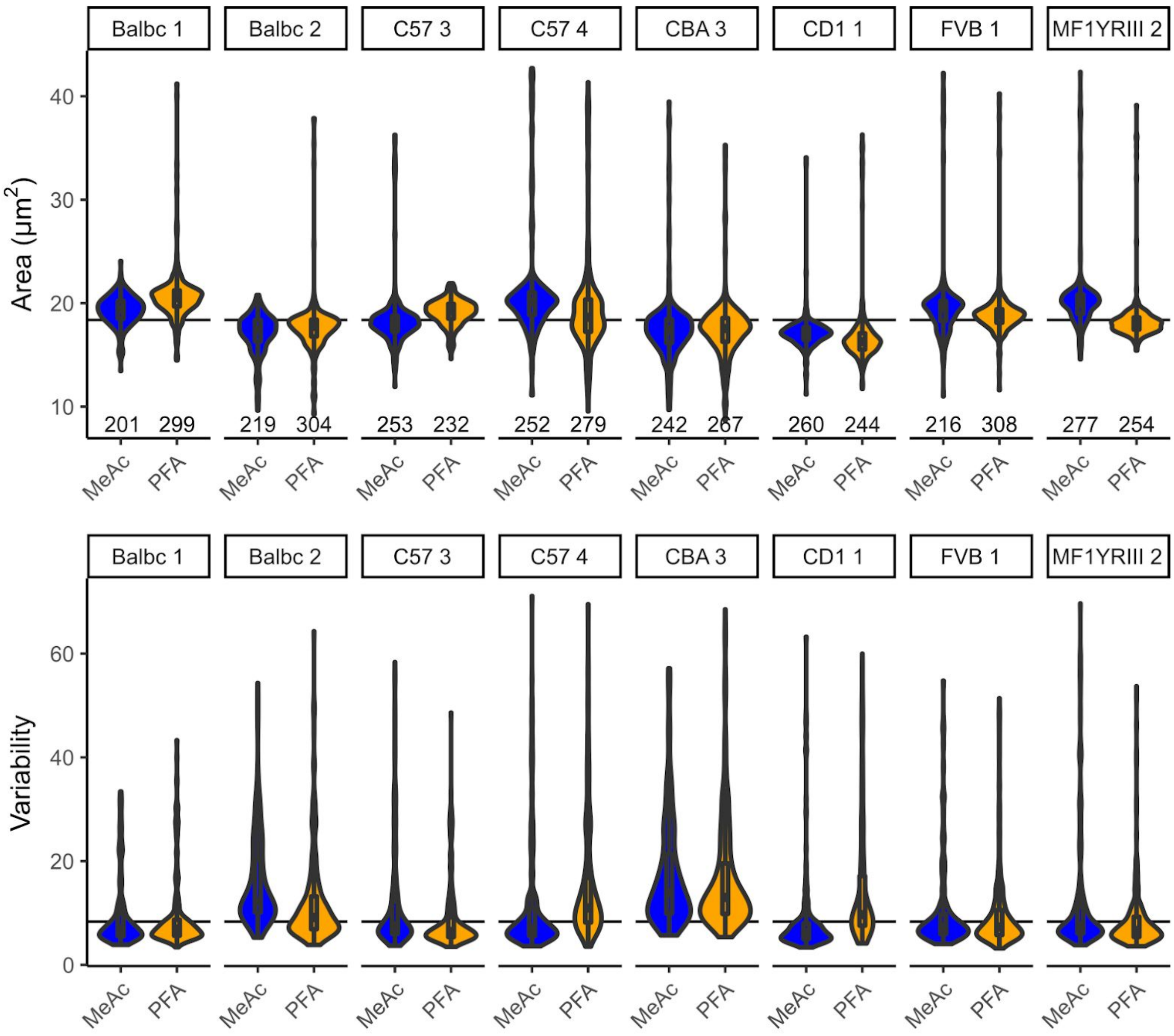

Supplementary Figure 8: Comparison of area and variability from Me:Ac and PFA fixed sperm shows slight differences by line, indicating comparisons should use consistent fixatives. Numbers of sperm are given in the upper panel.

Shape analysis is not specific to the microscope or camera used for imaging

We imaged nuclei from the same two C57Bl6 samples on four different microscope systems with different cameras, and compared the outputs (Supplementary Figure 9). Since the angle profiling method is based on a window size with a fixed proportion of the perimeter, the pixel scale of the microscope is independent of the profiles generated. The figure below shows the overlaid median and interquartile ranges for the imaged nuclei on each microscope. For the measured nuclear sizes, we found no significant differences in size after correction for the pixel/micron scale between the cameras, indicating that results will be comparable between different labs.

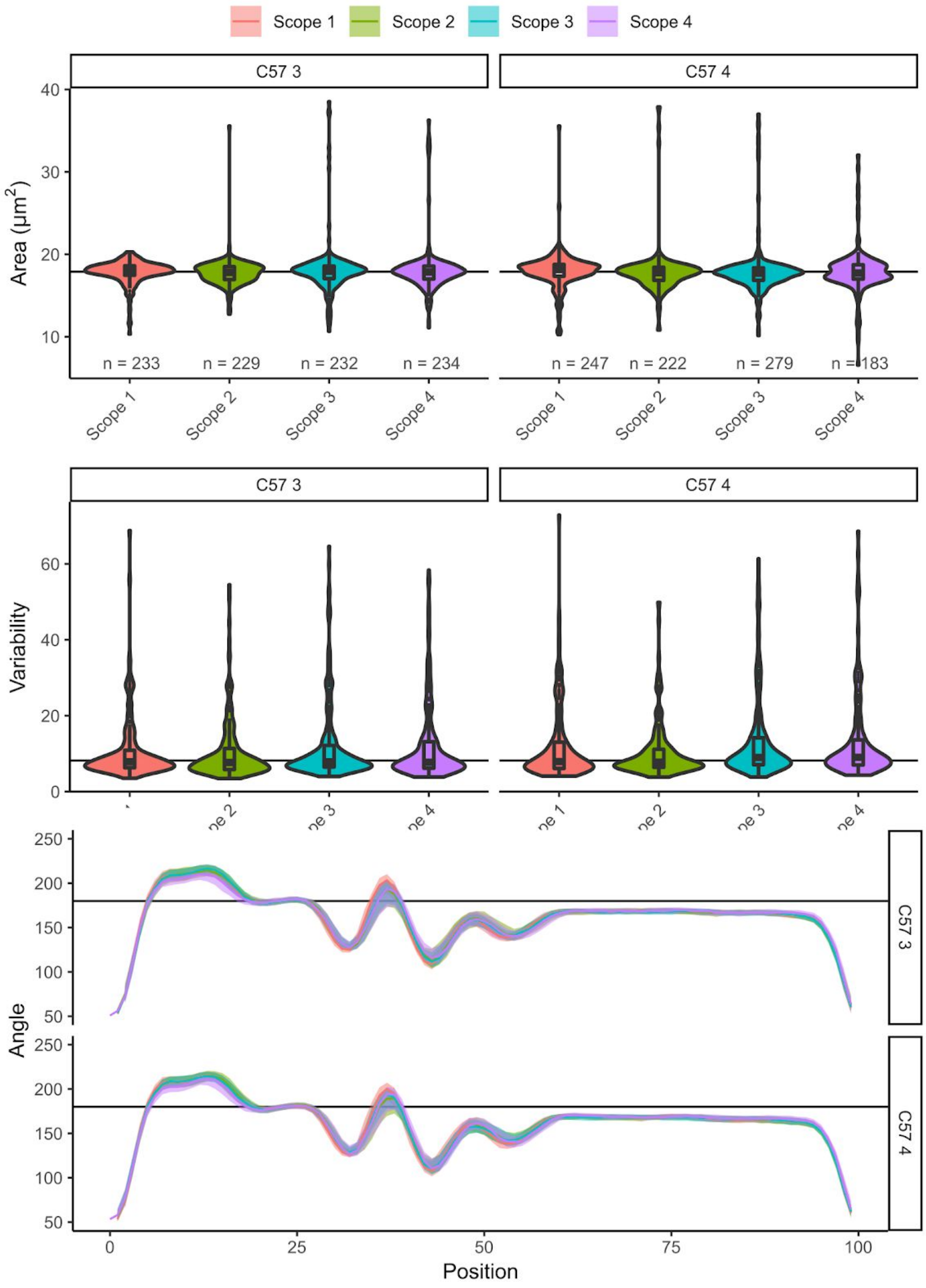

Supplementary Figure 9: Area, variability and angle profiles are not specific to the microscope used for imaging.

Automatic camera exposure times are sufficient for robust nucleus detection

We tested the effect of automatic camera exposure time versus fixed exposure times on samples (Supplementary Figure 10). We imaged sperm from ‘clean’ regions of the slide (where only well-separated nuclei are present) and from ‘dirty’ regions (where there were specks of dirt, cell clusters or other background features showing brightly in the field of view under DAPI fluorescence). The data show that the measured nuclear size and shape remains constant across a wide range of exposure times, and that the automatic exposure falls within this range.

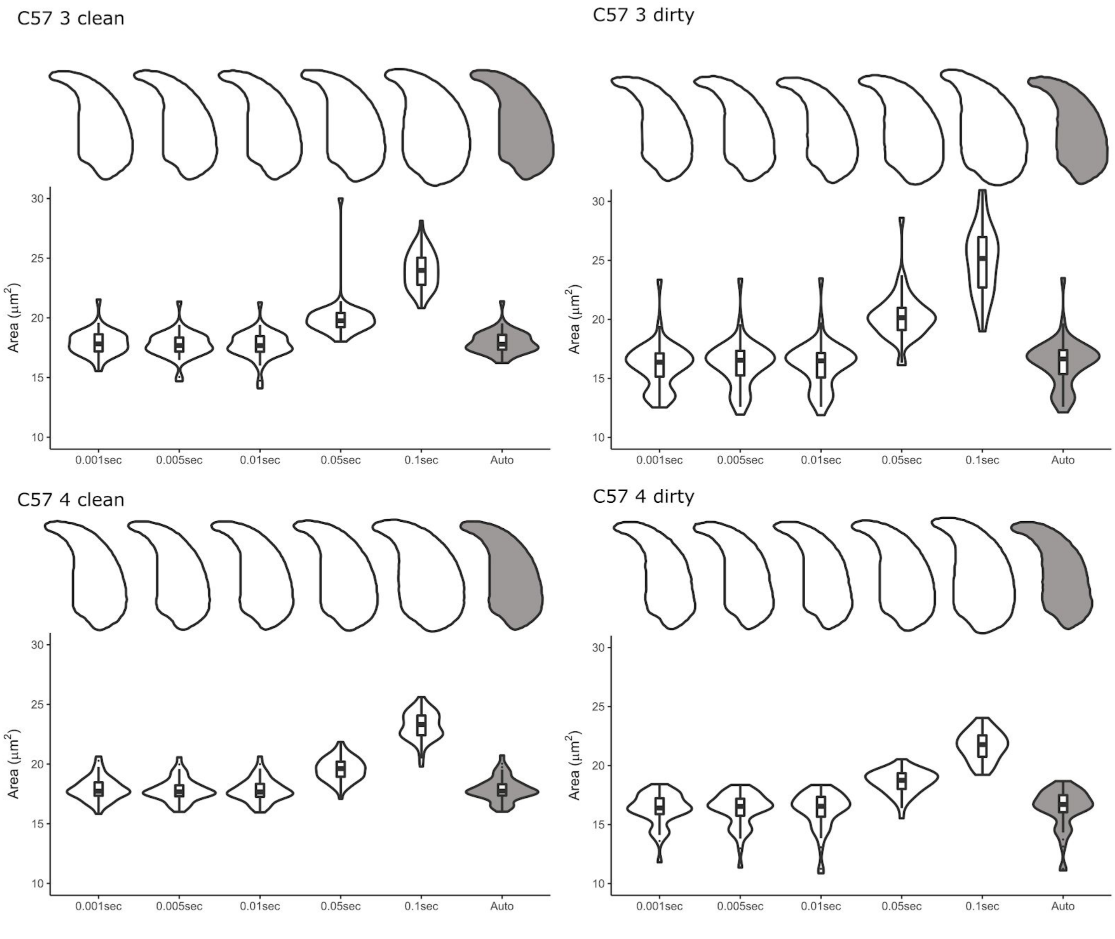

Supplementary Figure 10: Automatic exposure times provide consistent results, and are more convenient than setting fixed exposure time per sample.

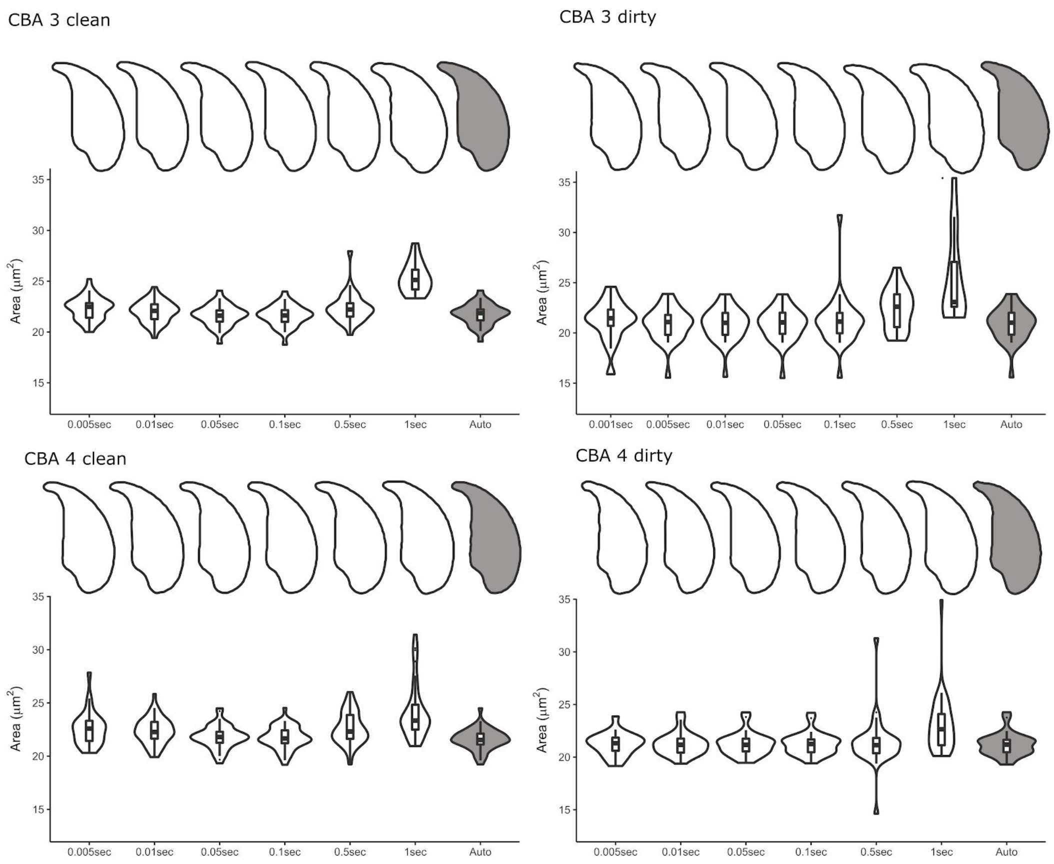

Supplementary Figure 10, continued: Automatic exposure times provide consistent results, and are more convenient than setting fixed exposure time per sample.

Segment lengths between C57Bl6 and CBA

Measurement of segment lengths between genotypes reveals the regions of greatest dissimilarity between the genotypes. For example, Supplementary Figure 11 shows the perimeter segments in CBA are longer than in C57Bl6 for all except the under-hook segment. CBAs and C57Bl6 have different segmentation patterns by default; we merged the two CBA segments making up the vertical region under the hook and combined the acrosomal and basal segments of C57Bl6 for simpler visualisation. The default segment patterns for strains are shown in Supplementary Figure 13.

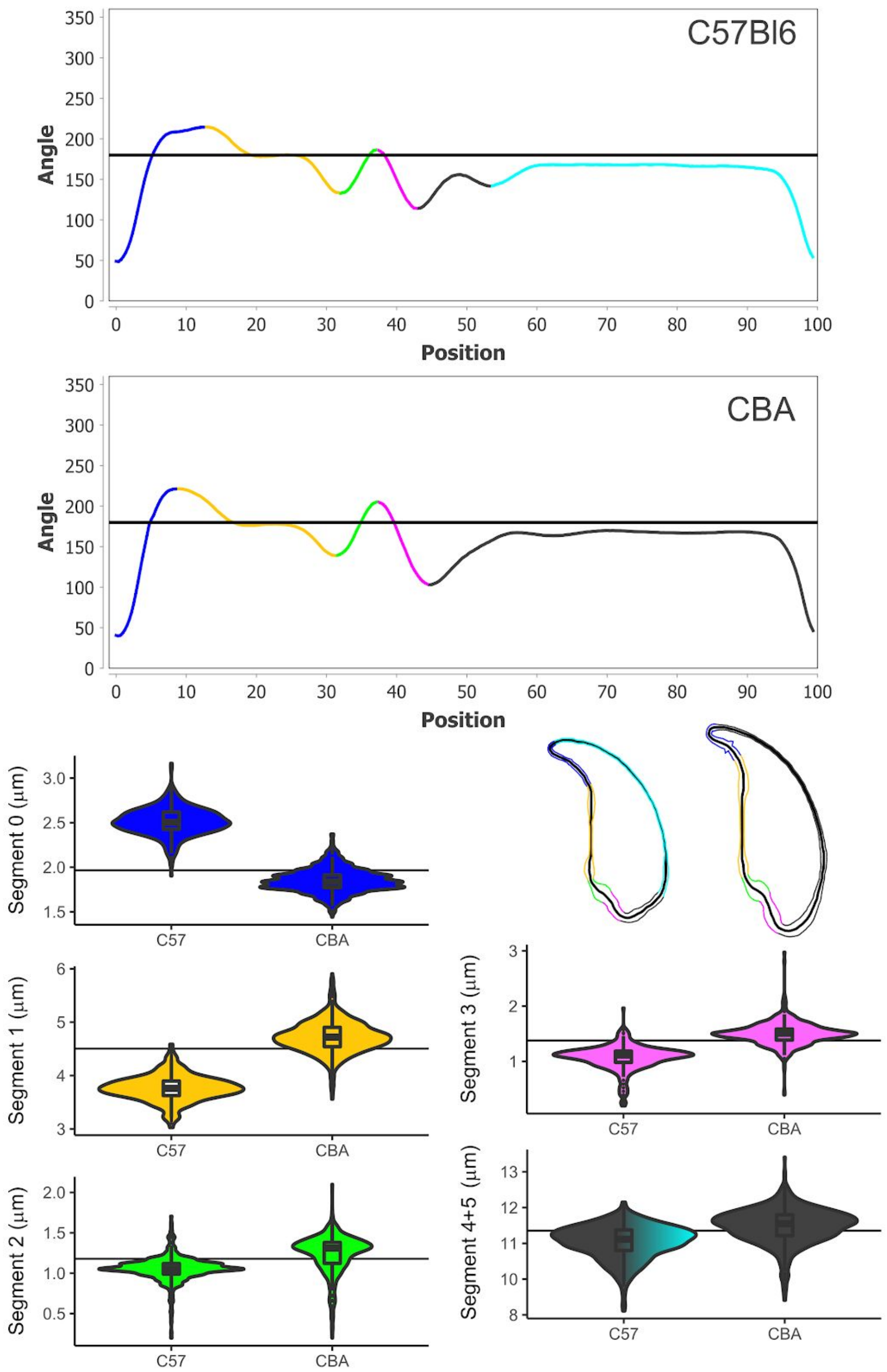

Supplementary Figure 11: Comparison of equivalent segment lengths between C57 and CBA. Segments 4 and 5 in C57 are combined, as there is no prominent dorsal angle in CBA.

Individual samples are consistent with strain averages

The individual sample data are shown for the strains presented in Supplementary Figure 12, and demonstrate that we are measuring strain-specific features, rather than individual sample specific features.

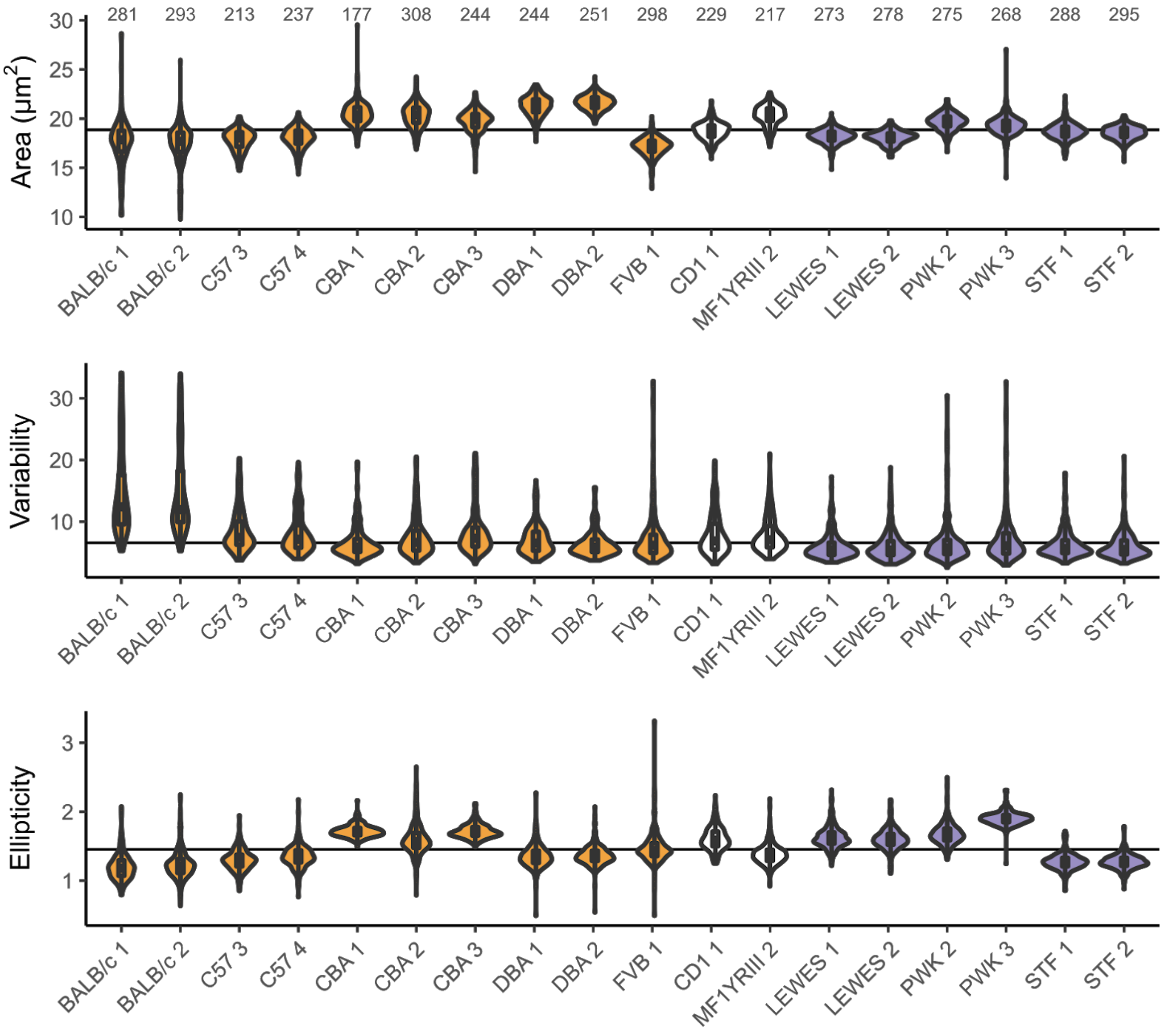

Supplementary Figure 12: The measured values for individual samples are consistent within strains, indicating inter-individual variability is a minimal concern. Samples are divided as in Fig 3: from left to right: inbred (yellow), outbred (white) and inbred wild-derived (blue). The number of sperm measured are given at the top of the upper panel.

Patterns of default profile segmentation varying by strain

Between five and seven Type II landmarks (local maxima or minima of curvature) were detected for each strain analysed, resulting in 5-7 "perimeter segments" for each strain. Since these landmarks themselves were variable between strains, the segmentation pattern in turn varies between strains.

The maximum number of landmarks was identified in *Mus spretus* (STF), these being:
1) The tip of the apical hook
2) The point of maximum indentation under the hook
3) A slight outward bulge on the ventral surface of the sperm
4) The sharp corner at the tail attachment site
5) The point of maximum indentation at the tail attachment site
6) The point of maximum curvature at the posterior end of the sperm head
7) A "dorsal angle" of increased positive curvature posterior to the acrosome

In the other strains, these landmarks were absent or relocated as follows:

- All laboratory strains except CBA and FVB lacked landmark 2, indicating that these strains have a flatter ventral surface from the hook all the way to the tail attachment.
- All strains except Balb/c, C57Bl6, FVB and MF1 lacked landmark 7, indicating that these strains have a more smoothly curved rear aspect to the sperm without a sharp dorsal angle.
- Balb/c and FVB have a pronounced narrowing of the sperm head around the tail attachment site, leading to effacement of the normal "socket" and loss of landmark 5
- In DBA, despite the lack of a clear dorsal angle, landmark 7 is relocated to the point of maximum curvature underlying the acrosome.

Next page:
Supplementary Figure 13: Default segmentation patterns for the strains in this study; segmentation pattern for each strain analysed (left), with the consensus nuclear outlines (right) showing the segments in the context of the cell shape for each strain. On the consensus nuclear outlines, the coloured lines indicate the local variability in the angle profile at each location around the nuclear perimeter.

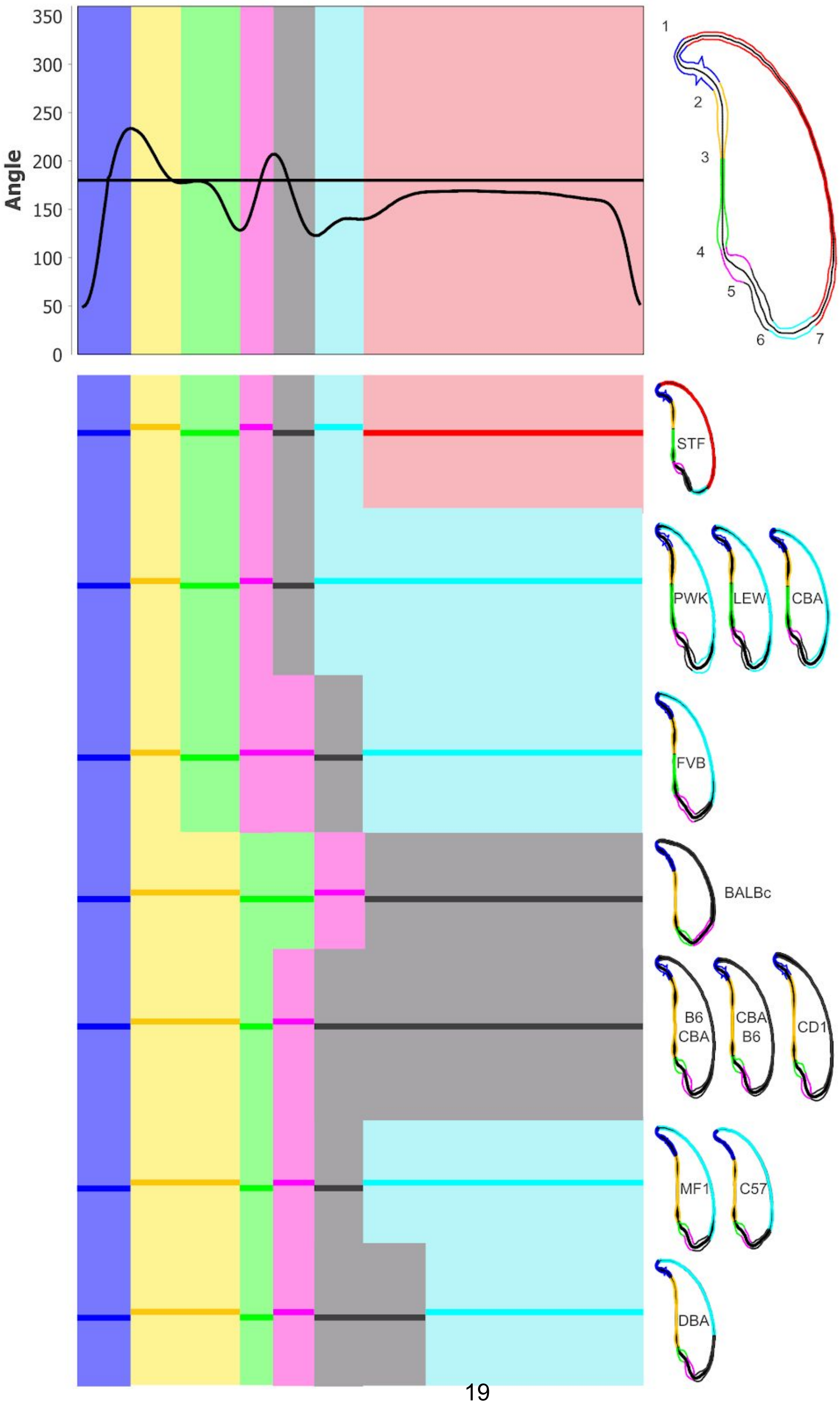
350
300
250
200
150
100
50
0
Angle
1
2
3
4
5
6
7
STF
PWK
LEW
CBA
FVB
BALBc
B6
CBA
CBA
B6
CD1
MF1
C57
DBA

Manual versus automated clustering

The accuracy of hierarchical clustering by shape was tested by clustering pooled samples from C57Bl6 and CBA. Since C57Bl6 and CBA sperm are slightly different sizes, the simplest partitioning of the mixed set is a binary cut-off at a given threshold for nuclear area. Passing the nuclear areas to a hierarchical clusterer and selecting the two most distinct clusters using the Ward clustering method was 83-85% accurate at separating the individual sperm by strain (Supplementary Table 4). To determine whether shape-based hierarchical clustering could improve upon this, we sampled values from the angle profile for each nucleus at regular intervals (corresponding to the original window proportion) and provided these as inputs to the clustering algorithm. The data show that adding the angle profiles provides an improvement on area, but adding further parameters has little effect. The reason for this is that the majority of the mis-clustered nuclei were slightly out of focus, or malformed.

**Cluster by area**	**Cluster 1 (712)**	**Cluster 2 (546)**
CBA (769)	83.49% (642)	16.51% (127)
C57Bl6 (490)	14.49% (71)	85.51% (419)
**Cluster by angle profile**	**Cluster 1 (724)**	**Cluster 2 (535)**
CBA (769)	91.42% (703)	8.58% (66)
C57Bl6 (490)	4.29% (21)	95.71% (469)
**Cluster by angle profile + ellipticity**	**Cluster 1 (738)**	**Cluster 2 (521)**
CBA (769)	92.85% (714)	7.15% (55)
C57Bl6 (490)	4.90% (24)	95.10% (466)
**Cluster by angle profile + ellipticity + feret + perimeter**	**Cluster 1 (693)**	**Cluster 2 (566)**
CBA (769)	89.47% (688)	10.53% (81)
C57Bl6 (490)	1.02% (5)	98.98% (485)

Supplementary Table 4: Clustering of pooled C57Bl6 and CBA images recovers original populations with >90% accuracy when using shape profiles, compared to ~85% accuracy when using only area. Additional shape parameters such as ellipticity yield minimal improvements in accuracy.

We compared the ability of humans with that of the clusterer. Three experienced assessors (BMS, CCR, PE) and five novice assessors (other PhD students and postdocs in the reproductive biology group at the University of Kent) assigned 100 nuclei as either C57Bl6 or CBA. Fifty nuclei were selected from each genotype. These nuclei were chosen manually to have a typical range of contrast and focus. Example images were visible throughout the test, plus an overlay of the consensus nuclei to show the volunteers the key differences in shape. The order of nuclei was randomised for each test, and each nucleus was shown twice during the test to gauge consistency. The clusterer used only the shape profiles, to give the humans the best possible advantage; when both shape and size parameters were used as the inputs for the clustering algorithm, the nuclei from the two genotypes were separated with perfect accuracy. Clustering was equivalent to an experienced assessor, and far superior than a novice at separating the nuclei by genotype (Supplementary Figure 14).

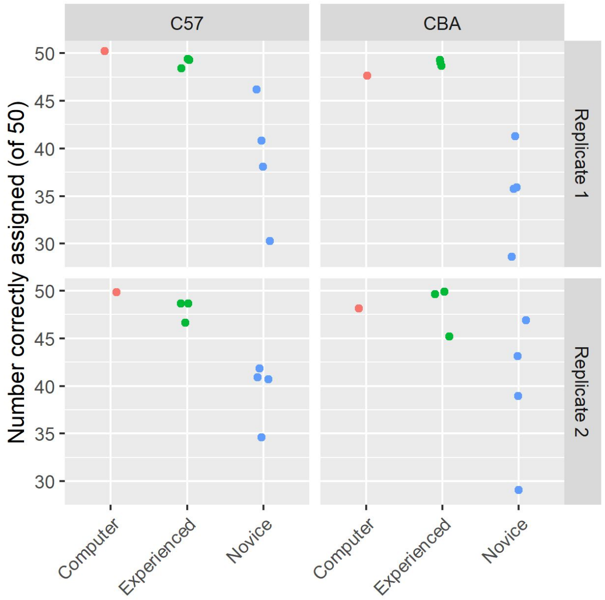

Supplementary Figure 14: Clustering separates genotypes as well as experienced human assessors, and better than novice assessors.

Comparison of our measurements with previous studies

The dimensions of entire sperm heads have been previously measured by Wyrobek et al in 1976 [6]. We compared our measurements for consistency, and found a good agreement between our measure of nuclear size and sperm head size, allowing for the thickness of the acrosome.

	**C57Bl6 - Wyrobek et al, 1976 [6]**	**C57Bl6 - This study**	**CBA - Wyrobek et al, 1976 [6]**	**CBA - This study**
**Bounding height (µm) and SEM**	7.61 ± 0.02	6.59 ± 0.02	8.64 ± 0.02	7.41 ± 0.02
**Body width (µm) and SEM**	3.54 ± 0.01	3.21 ± 0.01	3.51 ± 0.01	3.34 ± 0.01
**Area (µm2)**	20.91 ± 0.09	16.48 ± 0.04	22.34 ± 0.01	18.16 ± 0.05

Supplementary Table 5: Comparison of previous size measurements for whole sperm heads in CBA and C57Bl6 against our nuclear measurement shows consistency of measurement.

Correlation between our variability and the variability of other measured parameters

We compared our measure of variability within a population (calculated from the sum-of-squares difference between angle profiles) with the coefficient of variation (c.v.) of the other measured parameters (Supplementary Table 6). Our variability score correlates positively with variability in other measures. That is, individual samples that had a high c.v. for any given morphometric parameter not only tended to have high a c.v. for all other morphometric parameters measured, but also scored highly on our own sum-of-squares measure of variability.

**Parameter**	**Pearson correlation coefficient between the coefficient of variation of the parameter and the median variability**
Angle between reference points	0.93
Area	0.966
Aspect ratio	0.488
Bounding height	0.927
Bounding width	0.811
Circularity	0.928
Ellipticity	0.634
Elongation	0.83
Length of hook	0.373
Max feret	0.977
Min diameter	0.976
Perimeter	0.966
Regularity	0.857
Width of body	0.928

Supplementary Table 6: The Pearson correlation coefficients between the median variability measure and the coefficient of variation of other parameters per sample demonstrate our variability measure captures variation in nuclear shape across a range of parameters.

Clustered BALB/c nuclei match published phenotypic descriptions

The breakdown of nuclei by sample within our clusters from Figure 6 demonstrated we detected variation with the samples (Supplementary Table 7), and did not simply separate BALB/c 1 from BALB/c 2.

Cluster	Total sperm	Sperm from BALB/c 1	Sperm from BALB/c 2	Proportion from BALB/c 1
Total	574	281	293	48.95%
Cluster 1	117	60	57	51.28%
Cluster 2	281	137	144	48.75%
Cluster 3	131	58	73	44.27%
Cluster 4	45	26	19	57.78%

Supplementary Table 7: The BALB/c clusters divided by sample, demonstrating our clusters are not just detecting inter-individual variation

We applied the hierarchical clusterer on the hyper-condensed BALB/c nuclei in cluster 4, and discovered they fell broadly into two categories, similar to those described by Kishikawa et al [7]. Example nuclei from each cluster are shown below; the consensus averaging method becomes less effective with a small number of variable nuclei in each cluster.

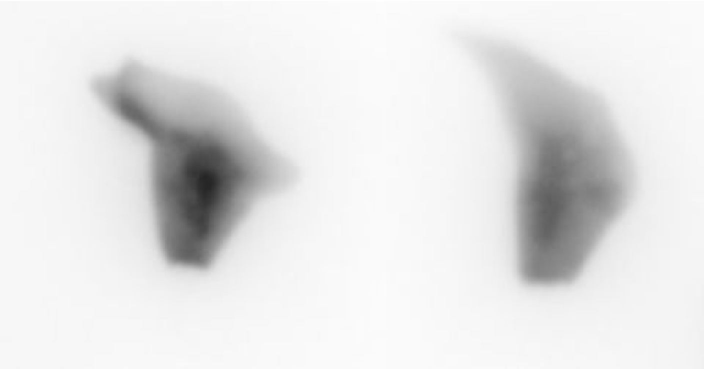

Supplementary Figure 15: Example nuclei from the two clusters of morphologically abnormal Balb/c sperm. DAPI channel images are inverted for clarity.

Supplementary References

1. Kuwahara M, Hachimura K, Eiho S, Kinoshita M. 1976 Processing of RI-Angiocardiographic Images. In Digital Processing of Biomedical Images, pp. 187–202. Springer, Boston, MA. (doi:10.1007/978-1-4684-0769-3_13)
2. Canny J. 1986 A computational approach to edge detection. Pattern Analysis and Machine Intelligence, IEEE Transactions on , 679–698.
3. Legland D, Arganda-Carreras I, Andrey P. 2016 MorphoLibJ: integrated library and plugins for mathematical morphology with ImageJ. Bioinformatics 32, 3532–3534. (doi:10.1093/bioinformatics/btw413)
4. Zahn, CT, Roskies, RZ. 1972 Fourier descriptors for plane closed curves. IEEE Transactions, Computers, C21: 269–281.
5. Bookstein FL. 1997 Morphometric Tools for Landmark Data: Geometry and Biology. Cambridge University Press.
6. Wyrobek AJ, Meistrich ML, Furrer R, Bruce WR. 1976 Physical characteristics of mouse sperm nuclei. Biophys. J. 16, 811–825. (doi:10.1016/S0006-3495(76)85730-X)
7. Kishikawa H, Tateno H, Yanagimachi R. 1999 Chromosome Analysis of BALB/c Mouse Spermatozoa with Normal and Abnormal Head Morphology. Biol Reprod 61, 809–812. (doi:10.1095/biolreprod61.3.809)
